# Commensal Pseudomonas fluorescens Strains Protect *Arabidopsis* from Closely Related Pseudomonas Pathogens in a Colonization-Dependent Manner

**DOI:** 10.1128/mbio.02892-21

**Published:** 2022-02-01

**Authors:** Nicole R. Wang, Christina L. Wiesmann, Ryan A. Melnyk, Sarzana S. Hossain, Myoung-Hwan Chi, Kitoosepe Martens, Kelly Craven, Cara H. Haney

**Affiliations:** a Department of Microbiology and Immunology, The University of British Columbia, Vancouver, British Columbia, Canada; b Noble Research Institute, Ardmore, Oklahoma, USA; University of Toronto

**Keywords:** *Arabidopsis*, microbiome, *Pseudomonas*, competitive exclusion, dysbiosis

## Abstract

Plants form commensal associations with soil microorganisms, creating a root microbiome that provides benefits, including protection against pathogens. While bacteria can inhibit pathogens through the production of antimicrobial compounds *in vitro*, it is largely unknown how microbiota contribute to pathogen protection *in planta*. We developed a gnotobiotic model consisting of Arabidopsis thaliana and the opportunistic pathogen Pseudomonas sp. N2C3, to identify mechanisms that determine the outcome of plant-pathogen-microbiome interactions in the rhizosphere. We screened 25 phylogenetically diverse Pseudomonas strains for their ability to protect against N2C3 and found that commensal strains closely related to N2C3, including Pseudomonas sp. WCS365, were more likely to protect against pathogenesis. We used comparative genomics to identify genes unique to the protective strains and found no genes that correlate with protection, suggesting that variable regulation of components of the core Pseudomonas genome may contribute to pathogen protection. We found that commensal colonization level was highly predictive of protection, so we tested deletions in genes required for *Arabidopsis* rhizosphere colonization. We identified a response regulator *colR*, and two ColR-dependent genes with predicted roles in membrane modifications (*warB* and *pap2_2*), that are required for Pseudomonas-mediated protection from N2C3. We found that WCS365 also protects against the agricultural pathogen Pseudomonas fuscovaginae SE-1, the causal agent of bacterial sheath brown rot of rice, in a ColR-dependent manner. This work establishes a gnotobiotic model to uncover mechanisms by which members of the microbiome can protect hosts from pathogens and informs our understanding of the use of beneficial strains for microbiome engineering in dysbiotic soil systems.

## INTRODUCTION

Plant pathogens are a large threat to global crop production, responsible for an estimated 30% of crop yield losses, causing economic losses and reducing global food security (1). While plants may encounter pathogens, they also exist in collaboration with beneficial microorganisms that colonize their tissues, forming microbiomes in ecological niches such as the phyllosphere (leaf surface) or rhizosphere (root surface). Due to the diverse collection of genes provided by each member of the rhizosphere microbiome, the rhizosphere microbiome has great genetic potential to beneficially impact the plant host, such as providing protection against pathogens. Plants are able to harness the genetic potential of the microbiome by shaping the community composition in response to changes in environmental conditions (2–4). The presence of specific beneficial bacteria, in turn, can protect against pathogens either directly, through antibiosis, or indirectly, by priming the plant immune system for future attack or by outcompeting pathogens for space and nutrients (5–7). As conventional farming practices or soil-less agriculture systems often result in plant growth in the absence of a natural rhizosphere microbiota, the risk of pathogen infection is likely heightened. While the genes and mechanisms that govern these beneficial plant-microbiome interactions are not fully understood, further research in biocontrol, plant immune system manipulation or evasion, or effective rhizosphere colonization could inform applications to engineer the microbiome to improve disease resistance (8).

Associations between *Arabidopsis* and Pseudomonas species can serve as model systems to study plant-microbe interactions in the rhizosphere (9–11). Pseudomonas fluorescens and related species are widely plant-associated and are ubiquitously present in the rhizosphere microbiome. Pseudomonas are enriched in disease suppressive soils (12) and are known to produce diverse antimicrobial compounds (13). The inoculation of single strains of P. fluorescens under gnotobiotic conditions has previously been used to identify strains or genes that are sufficient to provide benefits to the plant, such as promoting root growth, or acting as a biocontrol agent (14, 15). The ability to cultivate Pseudomonas strains with *Arabidopsis* under gnotobiotic conditions makes this genus useful in a reductionist approach to studying beneficial plant-microbe interactions (9).

The species complex P. fluorescens is genetically and functionally diverse, despite being over 97% identical based on 16S rRNA gene sequence (16, 17). Even within closely related P. fluorescens strains, host-associated lifestyles can differ. For example, the Brassicacearum clade of Pseudomonas contains the pathogenic Pseudomonas sp. N2C3 among numerous plant growth-promoting bacteria such as Pseudomonas sp. WCS365 and P. brassicacearum NFM421 (16). Pseudomonas sp. N2C3 can cause disease and stunting of plants within the Brassicaceae (including *Arabidopsis*, broccoli, kale) and Papaveroideae (poppy) families when grown in gnotobiotic conditions (16). However, when inoculated in natural soil, N2C3 was unable to stunt *Arabidopsis* growth (3). This suggests that N2C3 is an opportunistic pathogen and the presence of beneficial microbes in the rhizosphere may lead to protection against N2C3 pathogenesis. While other models exist to study microbial protection against plant pathogens, they have largely focused on leaf pathogens (18, 19); thus our system involving N2C3 and Pseudomonas strains provides a unique opportunity to understand how members of the rhizosphere microbiome can protect against pathogens.

Using Pseudomonas sp. N2C3 pathogenesis of *Arabidopsis* as a model system, we screened genome-sequenced commensal Pseudomonas for protection against this opportunistic pathogen. Closely related Pseudomonas strains with markers of both commensal and pathogenic lifestyles have been identified within the microbiome of the same plant (16, 20) indicating they naturally cooccur. By coinoculating Pseudomonas strains in a gnotobiotic system, we are able to model interactions that may occur between the plant, commensal microbiota, and pathogens. We identified a number of Pseudomonas strains that are close relatives of N2C3 that can protect against pathogenesis and used this model to identify genetic mechanisms that shape plant-pathogen-microbiome interactions.

## RESULTS

### Closely related Pseudomonas strains protect from an opportunistic pathogen.

We previously observed that the Pseudomonas pathogen N2C3 readily causes disease under gnotobiotic conditions (16) but fails to cause disease in soil (3), suggesting that members of the microbiome may protect against pathogenesis. To test this, we used Pseudomonas sp. N2C3 and *Arabidopsis* as a model to identify bacterial strains that are protective against N2C3 pathogenesis in the rhizosphere under gnotobiotic conditions. In preliminary studies, we tested two phylogenetically distinct Pseudomonas strains, WCS365 and CH267 for their ability to protect against N2C3 pathogenesis. While we found that WCS365 could protect against N2C3 at a 1:1 or 5:1 ratio (Fig. S1) as indicated by an increase in fresh weight, we found that CH267 failed to protect, even at a 5:1 ratio. As a result, we chose a 5:1 ratio for this screen to more robustly distinguish nonprotective strains from intermediately protective strains.

10.1128/mBio.02892-21.1 FIG S1 Protective ability of Pseudomonas strains coinoculated with N2C3 at 5:1 and 1:1 ratios differs between strains. (A) WCS365 can protect *Arabidopsis* when coinoculated with N2C3 at either a 5:1 or 1:1 ratio. (B) CH267 cannot protect *Arabidopsis* when coinoculated with N2C3 at either a 5:1 or 1:1 ratio. (A–B) Statistics were calculated by one-way ANOVA and Tukey’s HSD. Letters denote significance (*P* < 0.05). (C) Representative images of data quantified in (A) and (B). Download FIG S1, PDF file, 2.0 MB. Copyright © 2022 Wang et al. 2022 Wang et al. https://creativecommons.org/licenses/by/4.0/ This content is distributed under the terms of the Creative Commons Attribution 4.0 International license.

**FIG 1:**
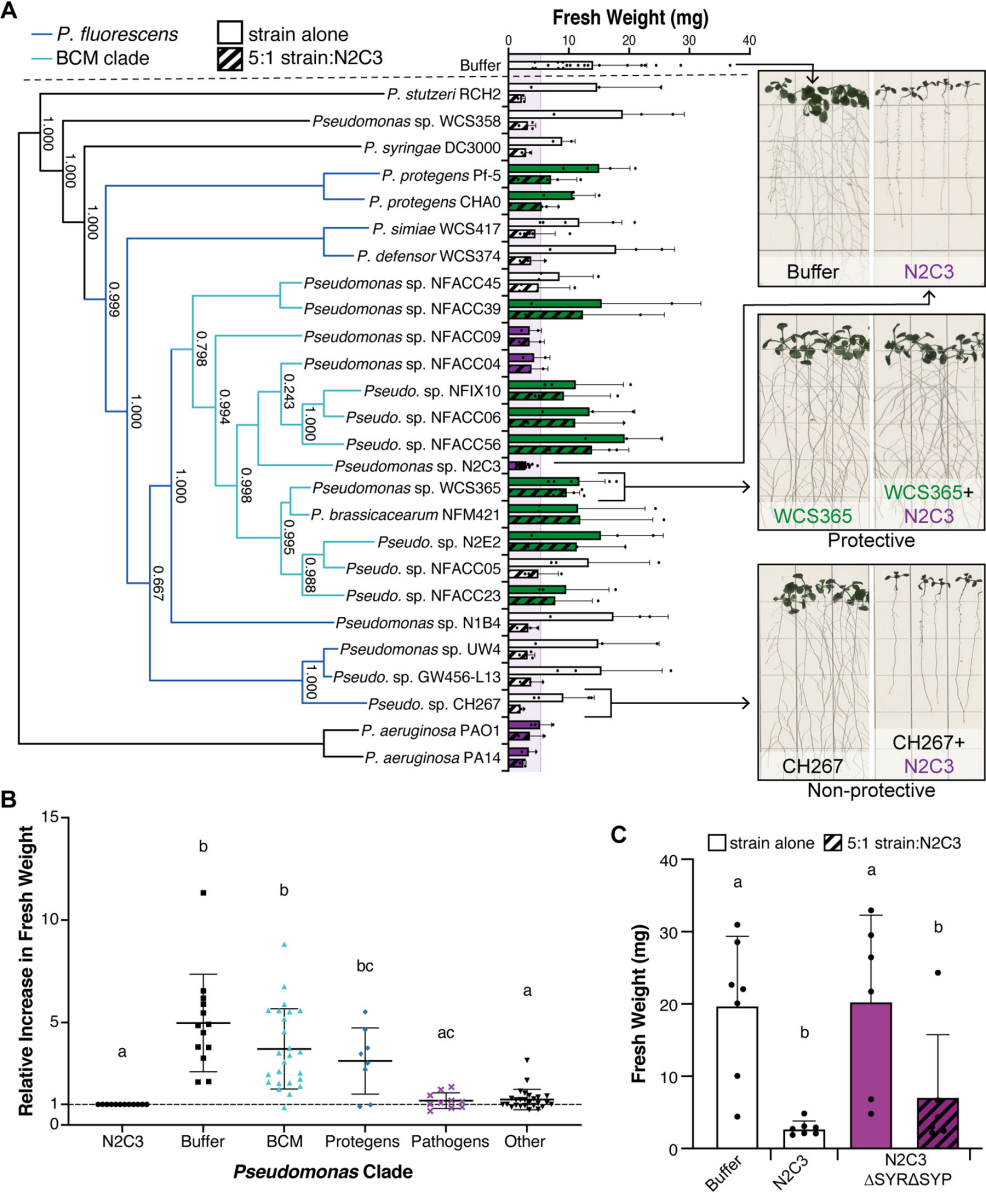
Phylogenetically related commensal Pseudomonas can protect against the Pseudomonas pathogen N2C3. (A) The ability to protect against N2C3 (green bars) is prevalent within the Brassicacearum/Corrugata/Mediterranea (BCM) clade (turquoise). Beneficial Pseudomonas strains outside the BCM clade, with the exception of Protegens strains Pf-5 and CHAO, are unable to protect (white bars). Pseudomonas strains were coinoculated with N2C3 at a 5:1 ratio. Fresh weight of *Arabidopsis* seedlings was used as a proxy for protection against N2C3. Pathogenic strains that stunt plant growth alone are marked in purple. Dashed line represents the mean + 3 standard deviations of N2C3-treated plants, estimating where 99.7% of measurements should fall under a normal distribution. Means that surpass this threshold were classified as protective (green bars). Representative images of buffer, pathogen (N2C3), a protective strain (WCS365) and a nonprotective strain (CH267) are shown. (B) Strains within the BCM and Protegens clades significantly increase plant fresh weight when coinoculated with N2C3, while nonpathogenic strains in other clades do not. Plant fresh weights were normalized by dividing by the average fresh weight of N2C3-treated control plants for the experiment. Letters indicate *P* < 0.05 as determined by one-way ANOVA and Tukey HSD. (C) Avirulent Pseudomonas sp. N2C3 cannot protect against virulent N2C3. A strain with a deletion of the pathogenicity island encoding syringomycin and syringopeptin (N2C3ΔSYRΔSYP) was added at a 5:1 ratio with virulent N2C3. (A-C) Each data point represents an average of 4–5 plants coinoculated with the test Pseudomonas strain and N2C3 from a single experiment. All repeated treatments tested in independent experiments were included as separate data points. Statistics were calculated using a one-way ANOVA and Tukey’s HSD, with letters indicating significance where *P* < 0.05. Lines represent mean +/− SD.

As the Pseudomonas genus is composed of genetically diverse species that are often present in the rhizosphere microbiome, 25 strains spanning the diversity of the Pseudomonas genus were tested for their ability to protect against N2C3 (Table S1A). We identified 10 Pseudomonas strains that when coinoculated at a 5:1 ratio with N2C3 increased the fresh weight of *Arabidopsis* (Fig. 1A; Fig. S2). We also identified 11 strains that failed to protect against N2C3 at a 5:1 ratio, as indicated by a similar fresh weight as seedlings treated with N2C3 alone (Fig. 1A; Fig. S2). An additional 4 strains stunted plants, including two P. aeruginosa strains known to be pathogenic on plants (21, 22), and two P. fluorescens*-*clade stains that have a pathogenicity island encoding syringomycin and syringopeptin, indicating they are pathogenic (16). Collectively this indicates that commensal Pseudomonas can protect from an opportunistic pathogen under gnotobiotic conditions and that there is natural variation in the protective ability across the genus Pseudomonas.

10.1128/mBio.02892-21.2 FIG S2 Representative images of *Arabidopsis* plants coinoculated with Pseudomonas strains and N2C3 at a 5:1 ratio. Representative images of data quantified in Fig. 1A, screening for Pseudomonas strains that could protect against N2C3. Download FIG S2, PDF file, 0.7 MB. Copyright © 2022 Wang et al. 2022 Wang et al. https://creativecommons.org/licenses/by/4.0/ This content is distributed under the terms of the Creative Commons Attribution 4.0 International license.

10.1128/mBio.02892-21.10 TABLE S1 Strains, mutants, and primers used in this study. Download Table S1, DOCX file, 0.05 MB. Copyright © 2022 Wang et al. 2022 Wang et al. https://creativecommons.org/licenses/by/4.0/ This content is distributed under the terms of the Creative Commons Attribution 4.0 International license.

We noted that 8 of the 10 protective Pseudomonas strains are within the Brassicacearum, Corrugata, and Mediterranea (BCM) clade of P. fluorescens (16) and that the other two are within the Protegens clade (Fig. 1A), suggesting that there may be some phylogenetic groups of Pseudomonas that are more able to protect against N2C3 pathogenesis than others. Using the data shown in Fig. 1A, we tested whether strains in the BCM and Protegens clade were significantly more likely to protect than other strains. We found a significant increase in *Arabidopsis* weights when seedlings were treated with strains from either the BCM or Protegens clades, but not other commensal Pseudomonas strains (Fig. 1B) supporting that these phylogenetic groups are more likely to be protective against N2C3.

Because of the strong phylogenetic signature of protection within the BCM clade, we wondered if an avirulent Pseudomonas sp. N2C3 mutant (a BCM strain) would be able to protect against virulent wild-type N2C3. N2C3 causes disease through quorum signaling-dependent synthesis of the nonribosomal peptides syringomycin (SYR) and syringopeptin (SYP) (16). We tested an avirulent N2C3 mutant with a deletion of SYR and SYP for its ability to protect against the wild type N2C3. Consistent with previous findings, the ΔSYRΔSYP mutant was no longer pathogenic (16); however, it was unable to protect against pathogenic N2C3 (Fig. 1C). These data suggest that mechanisms responsible for protection against N2C3 are uniquely present in the nonpathogenic BCM strains.

### Gain and loss of components of the Pseudomonas accessory genome does not explain protection against Pseudomonas sp. N2C3.

A number of phenotypes within Pseudomonas, ranging from plant-beneficial traits (16, 17), to virulence toward plants and animals (16, 23), can be attributed to the gain and loss of components of the Pseudomonas accessory genome. To identify genes that may correlate with protection, we used PyParanoid, a previously described comparative genomics platform (16), to identify genes that were unique to protective strains. To test whether Pseudomonas protegens Pf-5 obtained genes responsible for protection in the BCM clade through horizontal gene transfer, we identified 14 genes that were present in 7 BCM strains (Pseudomonas spp. N2E2, NFACC23, NFACC39, NFACC45, NFIX10, NFM421, and WCS365) and Pf-5, but absent in 7 nonprotective strains outside the BCM clade (Pseudomonas spp. CH267, GW456-L13, N1B4, UW4, WCS358, WCS374, and WCS417) (Fig. S3A). Both protective and nonprotective BCM strains were included in this comparison, to prevent exclusion of genes that are differentially expressed within the BCM clade. 12 out of 14 of these genes were deleted in WCS365, but none of them had a significant impact on its ability to protect against N2C3 (Fig. S3B).

10.1128/mBio.02892-21.3 FIG S3 Comparative genomics analyses performed to identify genes common to BCM strains and Pf-5. (A) To test whether Pf-5 obtained genes responsible for protection in the BCM clade through horizontal gene transfer, we identified 14 genes that were present in 7 BCM strains and Pf-5 (blue labels), but absent in 7 nonprotective strains outside the BCM clade (red labels). Both protective and nonprotective BCM strains were included in this comparison to capture genes that may be differentially expressed. Gene groups were labeled by their locus tag in WCS365. (B) 12/14 of the genes identified were deleted in WCS365, but none of them had an impact on its ability to protect against N2C3. Data were normalized by dividing by average fresh weight of N2C3-inoculated plants. Letters indicate *P* < 0.05, calculated using a one-way ANOVA and Tukey’s HSD. Mean +/− SD is plotted. Download FIG S3, PDF file, 0.5 MB. Copyright © 2022 Wang et al. 2022 Wang et al. https://creativecommons.org/licenses/by/4.0/ This content is distributed under the terms of the Creative Commons Attribution 4.0 International license.

We then searched for genes present in seven protective BCM strains (*Pseudomonas* spp. NFM421, N2E2, WCS365, NFIX10, NFACC06, NFACC39, and NFACC56), the protective non-BCM strain Pf-5, but absent in N2C3. This approach yielded only 1 unique gene (group_03914), encoding a CinA family protein (Fig. S4A). However, this gene was subsequently found to be present in 9 nonprotective strains (Pseudomonas spp. NFACC05, NFACC45, N1B4, PA14, PAO1, RCH2, WCS358, WCS374, and WCS417) and absent in the protective strain Pseudomonas protegens CHA0, indicating that it is unlikely to underlie protection. This comparison suggests that the Protegens and BCM clades may use distinct genetic mechanisms or differential regulation of core genes for pathogen protection.

10.1128/mBio.02892-21.4 FIG S4 Comparative genomics analyses performed to identify genes unique to protective strains. (A) Using PyParanoid, we searched for genes present in seven protective BCM strains (NFM421, N2E2, WCS365, NFIX10, NFACC06, NFACC39, and NFACC56) and the protective non-BCM strain Pf-5 (blue labels), but absent in N2C3 (red label). This approach yielded only 1 unique gene (group_03914), encoding a CinA family protein. However, this gene was subsequently found to be present in 9 nonprotective strains (NFACC05, NFACC45, N1B4, PA14, PAO1, RCH2, WCS358, WCS374, and WCS417) and absent in the protective strain CHA0, indicating that it is unlikely to underlie protection. (B) A comparative genomics analysis between strains within the BCM clade yielded no genes unique to protective BCM strains. We compared 8 protective BCM strains (NFM421, N2E2, WCS365, NFIX10, NFACC23, NFACC39, NFACC06, and NFACC56) (blue labels) with 3 nonprotective BCM strains (NFACC45, NFACC05, and N2C3) (red labels). Using this approach, we identified no genes present in the protective BCM strains, but absent in all 3 nonprotective BCM strains. Download FIG S4, PDF file, 0.5 MB. Copyright © 2022 Wang et al. 2022 Wang et al. https://creativecommons.org/licenses/by/4.0/ This content is distributed under the terms of the Creative Commons Attribution 4.0 International license.

We repeated the comparative genomics analyses using just strains within the BCM clade, to determine if the presence of any gene groups were responsible for differences in protection ability within this clade. To identify genes unique to protective BCM strains, we searched for genes present in 8 protective BCM strains (NFM421, N2E2, WCS365, NFIX10, NFACC23, NFACC39, NFACC06, and NFACC56) but absent in 3 nonprotective BCM strains (NFACC45, NFACC05, and N2C3). Using this approach, we identified no genes unique to the protective BCM strains (Fig. S4B). To identify genes that are unique to nonprotective BCM strains, we conducted the opposite analysis, searching for genes that were present in 3 nonprotective BCM strains (NFACC45, NFACC05, and N2C3), but absent in 8 protective BCM strains (NFM421, N2E2, WCS365, NFIX10, NFACC23, NFACC39, NFACC06, and NFACC56). Using this approach, we identified 1 gene group encoding an aldo/keto reductase (group_06773) that was unique to nonprotective BCM strains (Fig. S5A). However, when the presence/absence of this gene group was plotted onto a tree spanning the diversity of the Pseudomonas genus, we observed that not all nonprotective strains outside the BCM clade possessed this gene group, indicating that the absence of this gene was unlikely to be responsible for protection in the BCM clade.

10.1128/mBio.02892-21.5 FIG S5 Comparative genomics analyses to identify genes absent from protective BCM strains. (A) To identify genes that are unique to nonprotective BCM strains, we searched for genes that were present in 3 nonprotective BCM strains (NFACC45, NFACC05, and N2C3) (blue labels), but absent (red labels) in 8 protective BCM strains (NFM421, N2E2, WCS365, NFIX10, NFACC23, NFACC39, NFACC06, and NFACC56). Using this approach, we identified 1 gene group encoding an aldo/keto reductase (group_06773) that was unique to nonprotective BCM strains. However, not all nonprotective strains outside the BCM clade possessed this gene group, indicating that the absence of this gene was unlikely to be responsible for protection in the BCM clade. (B) To identify genes whose absence may be responsible for a BCM clade-specific mechanism of protection, we searched for genes that were present in 9 non-BCM strains (Pf-5, CH267, GW456-L13, N1B4, RCH2, UW4, WCS358, WCS374, and WCS417) (blue labels) but absent in 7 protective BCM strains (N2E2, NFACC06, NFACC39, NFACC56, NFIX10, NFM421, and WCS365) (red labels). However, we were unable to identify any genes that met these criteria. Download FIG S5, PDF file, 0.5 MB. Copyright © 2022 Wang et al. 2022 Wang et al. https://creativecommons.org/licenses/by/4.0/ This content is distributed under the terms of the Creative Commons Attribution 4.0 International license.

To identify genes whose absence may be responsible for a BCM clade-specific mechanism of protection, we searched for genes that were present in 9 non-BCM strains (Pf-5, CH267, GW456-L13, N1B4, RCH2, UW4, WCS358, WCS374, and WCS417) but absent in 7 protective BCM strains (N2E2, NFACC06, NFACC39, NFACC56, NFIX10, NFM421, and WCS365) (Fig. S5B). However, we were unable to identify any genes that met this criterion. These data suggested that genes uniquely present or absent in the protective or nonprotective BCM clade members were not responsible for their ability to protect against N2C3.

**FIG 2:**
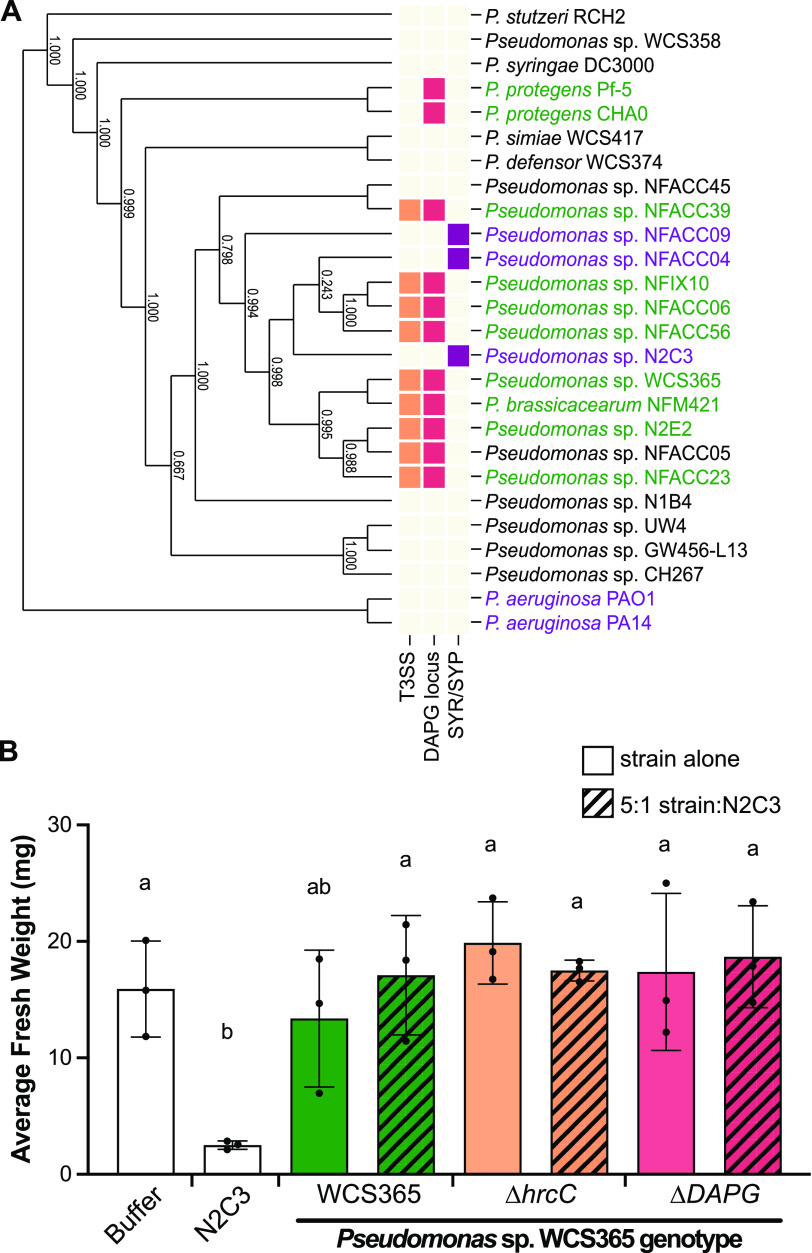
The ability to protect against N2C3 across the genus Pseudomonas is not due to horizontal transfer of components of the accessory genome. (A) Comparative genomics failed to identify any genes unique to protective strains (Fig. S3 and S4). A previous GWAS identified a type 3 secretion system (T3SS) and diacetylphloroglucinol (DAPG) biosynthesis genes that are anti-correlated with pathogenicity in the BCM clade (16). Strain names are color coded according to their ability to protect (green), not protect (black) or stunt plants alone (purple). (B) Pseudomonas sp. WCS365 *ΔhrcC* (deficient in T3SS) and ΔDAPG mutants still protect against N2C3. Letters indicate *P* < 0.05 as determined using a one-way ANOVA and Tukey’s HSD test. Each dot represents the average weight of 5 plants from a single biological replicate; separate dots are independent experiments with mean +/− SD plotted.

A previous genome-wide association study identified three genomic regions that are negatively correlated with pathogenicity within the BCM clade, and are typically only present in the nonpathogenic members of the clade (16). These genomic regions encode a type III secretion system (T3SS), diacetylphloroglucinol (DAPG) biosynthesis genes, and a T3SS effector, HopAA (16, 24) (Fig. 2A). To test whether T3SS or DAPG could contribute to protection against N2C3, we generated clean deletions of the *hrcC* gene (25) and DAPG biosynthesis island in the protective strain Pseudomonas sp. WCS365. We found that the Pseudomonas WCS365 Δ*hrcC* and ΔDAPG mutants had similar levels of protection as wild-type WCS365 (Fig. 2B, Fig. S6). As we were unable to identify components of the Pseudomonas accessory genome involved in protecting against N2C3 we instead hypothesized that regulation of core Pseudomonas genetic components, rather than presence/absence of genes, may be required for protection.

**FIG 3:**
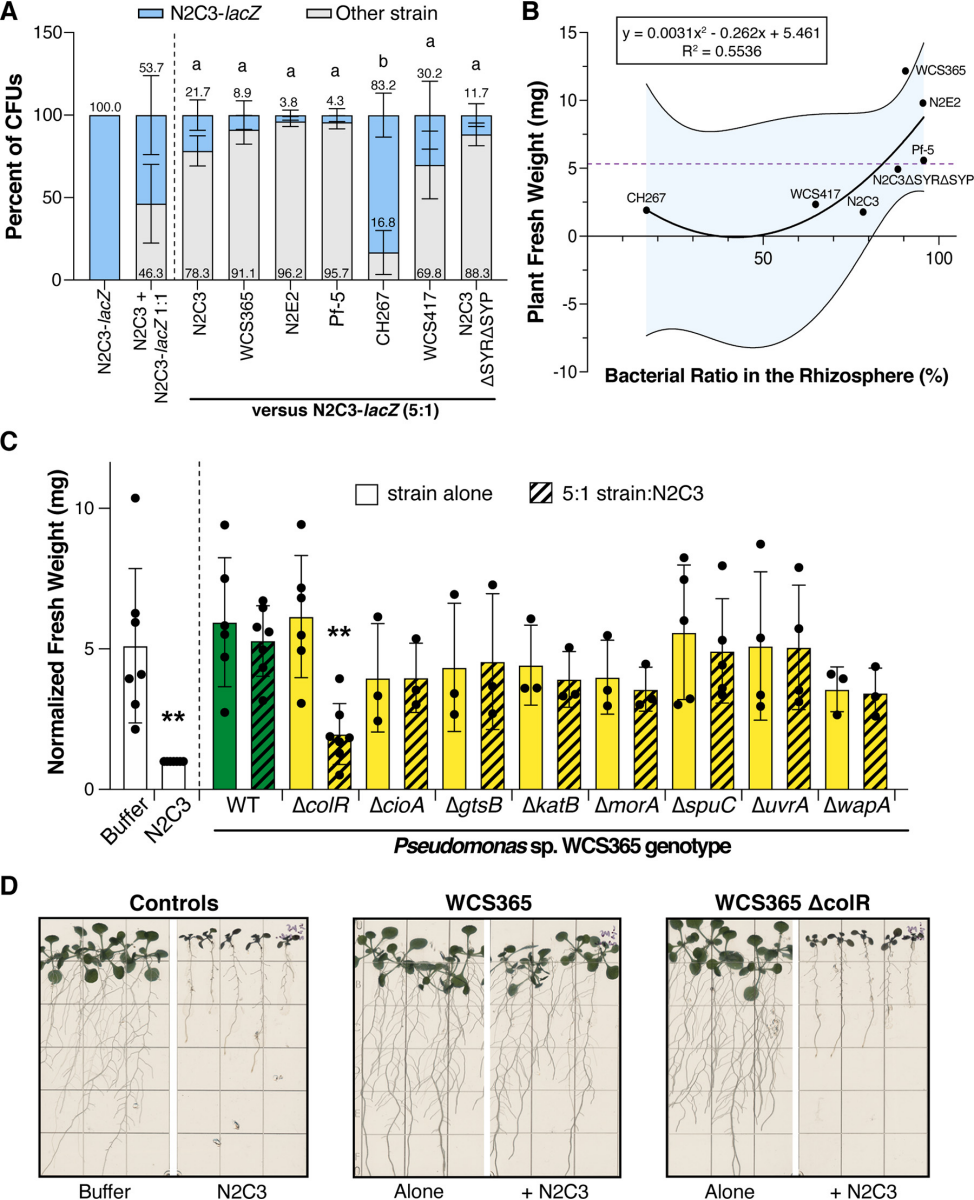
Protection against Pseudomonas sp. N2C3 is rhizosphere colonization dependent. (A) Pseudomonas strains that protected against N2C3, such as members of the BCM clade (WCS365, N2E2) and Pf-5, maintained at least a 5:1 ratio when in competition with N2C3, while strains that cannot protect had decreased fitness when coinoculated with N2C3. Pseudomonas strains were competed against a N2C3 transposon insertion mutant expressing *lacZ.* Relative abundance of each strain was determined by grinding whole plants, plating on media containing X-gal, and counting blue and white colonies. Letters denote significance (*P* < 0.05) by two-way ANOVA and Tukey HSD. Bars represent mean +/− SD. (B) Correlation between the bacterial fitness and the plant fresh weight. Data points represent the average fresh weights and average ratios for the strain coinoculated with N2C3-*lacZ*. Polynomial linear regression plotted with shaded area representing 95% confidence intervals. The purple dashed line denotes the upper 99.7% confidence interval for increase in plant weight when coinoculated with N2C3 as determined in Fig. 1 (C) Pseudomonas sp. WCS365 deletion mutants of genes previously shown to be required for colonization of the *Arabidopsis* rhizosphere. Only deletion of the response regulator *colR* resulted in a loss in ability of Pseudomonas sp. WCS365 to protect *Arabidopsis* from N2C3. Each strain was coinoculated onto roots with N2C3 at a 5:1 ratio. **P < 0.05 relative to WT WCS365 + N2C3 determined by one-way ANOVA and Tukey HSD. Mean +/− SD is plotted. (D) Representative images showing loss of protection by Pseudomonas sp. WCS365 *ΔcolR* mutant.

10.1128/mBio.02892-21.6 FIG S6 Representative images of *Arabidopsis* plants inoculated with Pseudomonas sp. WCS365 deletion mutants while screening for a loss of protection when coinoculated with N2C3 in a 5:1 ratio. Representative images of data quantified in Fig. 2B, 3C, and S3B. Download FIG S6, PDF file, 0.9 MB. Copyright © 2022 Wang et al. 2022 Wang et al. https://creativecommons.org/licenses/by/4.0/ This content is distributed under the terms of the Creative Commons Attribution 4.0 International license.

### Colonization ability predicts protection against Pseudomonas sp. N2C3.

To gain insights into how protective strains may protect *Arabidopsis*, we first tested whether the protective Pseudomonas sp. WCS365 could directly kill N2C3 or inhibit its virulence. Using a bacterial cross streak assay, we found that WCS365 did not inhibit growth of N2C3 (Fig. S7A). To test whether WCS365 can inhibit quorum signaling by N2C3, we used Chromobacterium violaceum CV026 as a biosensor for C4-C8 AHL molecules, since it reports AHLs from N2C3 through the production of the purple pigment violacein (16, 26). We found that violacein production was maintained when WCS365 and N2C3 were mixed and plated onto LB agar, indicating that WCS365 does not suppress N2C3 quorum signaling (Fig. S7B). These data indicate that WCS365 does not directly kill N2C3 nor does it interfere with regulation of SYR/SYP-production in N2C3 *in vitro*.

10.1128/mBio.02892-21.7 FIG S7 Pseudomonas sp. WCS365 does not inhibit growth or quorum signaling of N2C3 *in vitro*. (A) Overnight cultures of WCS365 and N2C3, grown in LB, were streaked onto LB agar. The first strain was streaked vertically, and the second strain was streaked horizontally at a 90° angle. Neither WCS365 nor N2C3 created a zone of inhibition that prevented the growth of the strain that was streaked second. (B) Although N2C3 virulence relies on quorum signaling genes (E.g. the AHL synthase *luxI*) present in a pathogenicity island in the BCM clade (16), the protective WCS365 strain does not protect through quorum quenching. N2C3 AHL production is detected by the production of violacein, a visible purple pigment, by Chromobacterium violaceum CV026. Each panel contains two streaks. On the left is the bacterial mixture described below each panel. On the right is the CV026 biosensor, which produces violacein in the presence of C4-C8 AHL molecules. Download FIG S7, PDF file, 2.6 MB. Copyright © 2022 Wang et al. 2022 Wang et al. https://creativecommons.org/licenses/by/4.0/ This content is distributed under the terms of the Creative Commons Attribution 4.0 International license.

Since we found that Pseudomonas sp. WCS365 does not kill N2C3 or inhibit quorum signaling *in vitro*, we hypothesized that protective strains may outcompete the pathogen *in planta*. To test this, we generated a N2C3-*lacZ* transposon insertion mutant to allow us to homogenize seedlings and the attached bacteria, and distinguish CFU from distinct Pseudomonas strains on media containing X-gal. We found that protective strains (WCS365, N2E2 and Pf-5), maintained at least a 5:1 ratio (similar to the initial inoculum) in competition with N2C3-*lacZ* (Fig. 3A). In contrast, CH267, which cannot protect *Arabidopsis* from N2C3, was significantly outcompeted by N2C3 (Fig. 3A). Absolute CFU counts showed that protective strains lowered both the number of N2C3-*lacZ* CFU present, as well as the total bacterial load (Fig. S8). We tested whether competition against N2C3 could predict protection in commensal Pseudomonas strains and found a polynomial linear relationship with a threshold for protection of approximately 5:1 (Fig. 3B). The model y = 0.0031 × ^2^ - 0.262× + 5.461 explains about 55% of the plant fresh weight in response to bacterial colonization levels in the rhizosphere. These data indicate that colonization may be a prerequisite for pathogen protection among Pseudomonas species strains, but that factors in addition to colonization may contribute to protection.

**FIG 4:**
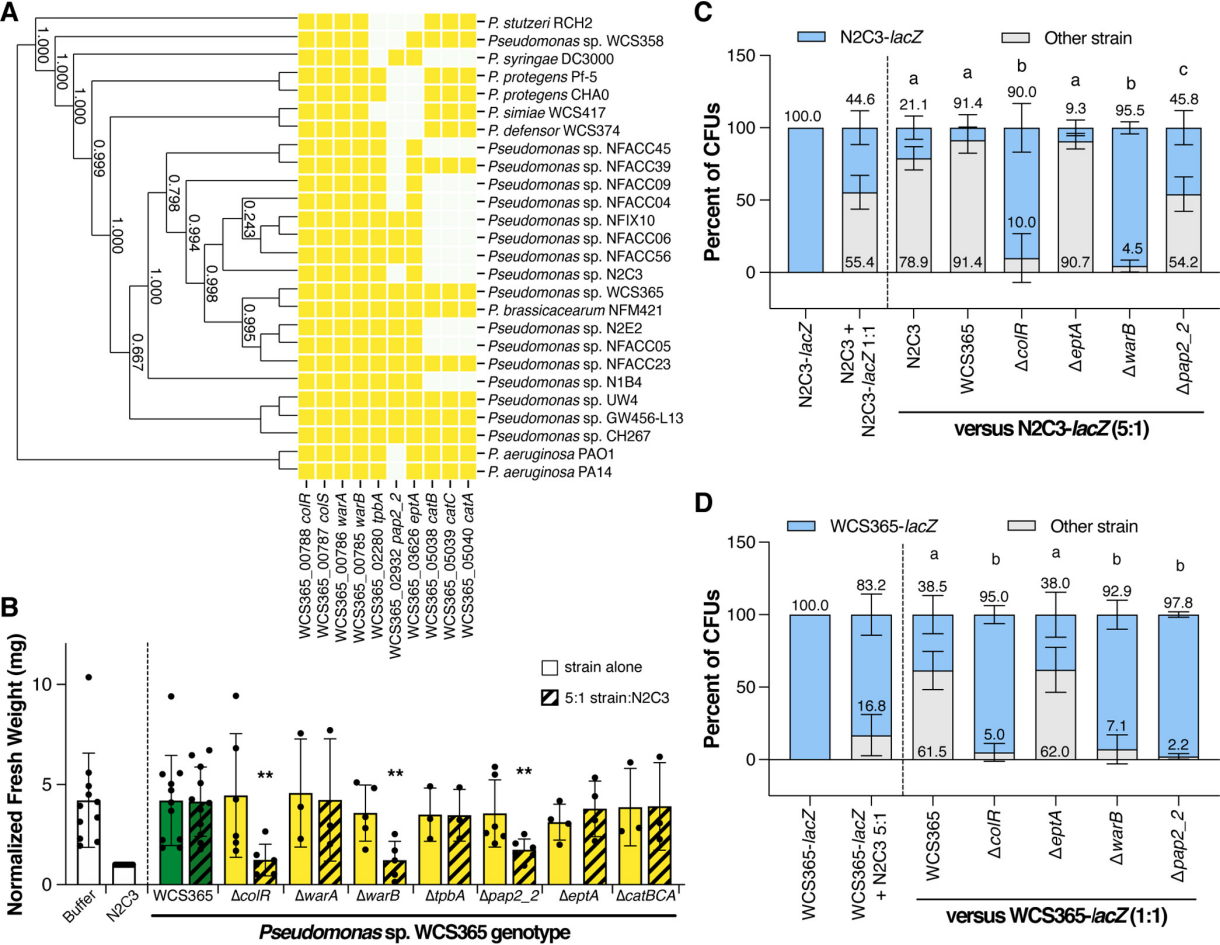
Protection against N2C3 is dependent on *colR, warB*, and *pap2_2*. (A) The two-component system ColR/S and eight previously identified genes whose expression is ColR/S dependent (31, 38) are largely conserved across both protective and nonprotective Pseudomonas strains. (B) Deletion mutants Pseudomonas sp. WCS365 Δ*warB* and Δ*pap2_2* were unable to protect against N2C3 when coinoculated at a 5:1 ratio. Data were normalized by dividing by average fresh weight of N2C3-inoculated control plants. **P < 0.05 by one-way ANOVA and Dunnett’s *post hoc* test. Data for WCS365 strains inoculated alone were excluded from statistical analyses. (C) Deletion of the ColR-regulated genes, *warB* and *pap2_2*, but not *eptA*, results in significantly decreased fitness in competition with N2C3 and (D) When competed against N2C3-*lacZ* in a 5:1 ratio and WCS365-*lacZ* at a 1:1 ratio, nonprotective WCS365 deletion mutants (Δ*colR*, *ΔwarB*, and Δ*pap2_2*) were significantly outcompeted. Letters denote significance (*P* < 0.05) by two-way ANOVA and Tukey’s HSD.

10.1128/mBio.02892-21.8 FIG S8 Absolute CFU counts of LacZ assays quantifying competition between Pseudomonas strains and N2C3. (A) Absolute CFU counts of Pseudomonas strains inoculated in competition with N2C3-*lacZ* in a 5:1 ratio, used to calculate relative abundances plotted in Fig. 3A. Statistics were conducted by one-way ANOVA and Dunnett’s multiple-comparison test. *P < 0.05 indicate CFU counts of the LacZ strain (blue) relative to N2C3-*lacZ* alone. (B) Absolute CFU counts of WCS365 deletion mutants coinoculated with N2C3-*lacZ* in a 5:1 ratio. These include protective and nonprotective Pseudomonas strains, WCS365 deletion mutants in *colR*-regulated genes, and WCS365 deletion mutants in candidate genes obtained through comparative genomics. These data were also used to calculate the relative abundance plotted in Fig. 4C. Statistics were conducted by one-way ANOVA and Dunnett’s multiple comparison’s test. *P < 0.05 denote differences in N2C3-*lacZ* CFUs relative to when coinoculated with WCS365 at a 5:1 ratio. (A-B) Total CFU counts (LacZ strain + other strain) were also analyzed by one-way ANOVA and Dunnett’s multiple-comparison test, but there were no significant differences (*P* < 0.05) between treatments. Bars represent mean + SD. (C) Change in N2C3-*lacZ* CFUs when plants were coinoculated with protective or nonprotective strains/mutants and N2C3-*lacZ* at a 5:1 ratio. (D) Change in total bacterial load when plants were coinoculated with protective or nonprotective strains/mutants and N2C3-*lacZ* at a 5:1 ratio. (C-D) Letters denote *P* < 0.05, calculated by one-way ANOVA and Tukey’s HSD. Download FIG S8, TIF file, 14.7 MB. Copyright © 2022 Wang et al. 2022 Wang et al. https://creativecommons.org/licenses/by/4.0/ This content is distributed under the terms of the Creative Commons Attribution 4.0 International license.

Since we found that colonization was correlated with protection against Pseudomonas sp. N2C3 (Fig. 3B), we hypothesized that competitive exclusion could be important for protection. During competitive exclusion, existing members of the microbiome protect against pathogens by competing for space and nutrients rather than direct antagonism (27). To test whether protection from N2C3 is colonization dependent, we tested 8 WCS365 deletion mutants that were found to have fitness defects in a previous Tn-Seq screen (28) for their ability to protect against N2C3. We found that the majority of genes tested are broadly conserved across protective and nonprotective strains (Fig. S9). Of the mutants tested, we found that only WCS365 Δ*colR* lost its ability to protect against N2C3 (Fig. 3C). ColR is a response regulator in a two-component system that has previously been shown to be necessary for colonization of plant roots (28, 29). This indicates that protection against N2C3 relies on a ColR-dependent mechanism for colonization.

10.1128/mBio.02892-21.9 FIG S9 Distribution of colonization genes across Pseudomonas spp. Previously identified genes involved in colonization and rhizosphere fitness (28) are largely conserved across both protective and nonprotective Pseudomonas strains. Download FIG S9, PDF file, 0.4 MB. Copyright © 2022 Wang et al. 2022 Wang et al. https://creativecommons.org/licenses/by/4.0/ This content is distributed under the terms of the Creative Commons Attribution 4.0 International license.

ColR/S is a rhizosphere colonization or fitness determinant of Pseudomonas in both *Arabidopsis* and potato (28, 29). Several genes expressed in a ColR/S-dependent manner have been identified, including several required for rhizosphere colonization. These include an operon adjacent to ColR/S that includes *warA* (encoding a putative methyltransferase) and *warB* (encoding a putative heptose kinase), which are predicted to modify bacterial lipopolysaccharide (LPS) ([38]; note: WCS365 *warA* and *warB* were previously called *orf222* and *wapQ* respectively, but were renamed here due to orthology to *P. aeruginosa* PAO1 *warA* and *warB*). Additionally, ColR/S in P. putida regulates expression of *eptA*, encoding a phosphoethanolamine (pEtN) transferase, which adds a pEtN to LPS (30). A recent RNAseq experiment identified genes in WCS365 that were induced in the rhizosphere in a ColR-dependent manner (31). These included *pap2_2*, predicted to encode a phosphatidic acid phosphatase, which could affect membrane lipid homeostasis (32), *tpbA*, predicted to encode a tyrosine phosphatase, which controls extracellular DNA levels and biofilm formation in P. aeruginosa (33), and *catB/C/A*, predicted to encode catalase enzymes that detoxify reactive oxygen species (34). We found that these genes have variable conservation across the Pseudomonas genus but are present in both protective and nonprotective strains (Fig. 4A). We generated deletion mutants in Pseudomonas sp. WCS365 *warA*, *warB*, *tpbA*, *pap2_2*, *eptA*, and *catBCA* and tested the mutants for protection against N2C3. We found that similar to the *ΔcolR* mutant, the WCS365 Δ*warB* and *Δpap2_2* mutants failed to protect against N2C3 (Fig. 4B). In contrast, the Δ*warA*, Δ*tpbA*, *ΔeptA*, and Δ*catBCA* mutants retained their ability to protect against N2C3. These results indicate that a subset of ColR-dependent genes are required for protection against N2C3.

To test whether *ΔcolR*, *ΔwarB*, and Δ*pap2_2* mutants had competition defects in the rhizosphere, we competed them against N2C3-*lacZ* and WCS365-*lacZ*. We found that protective strains WCS365 and WCS365Δ*eptA* maintained their inoculated ratios in the rhizosphere in competition with either N2C3 or WCS365 (Fig. 4C and D), whereas the nonprotective strains WCS365 Δ*colR*, Δ*warB*, and *Δpap2_2* were significantly out-competed by N2C3 (Fig. 4C) and WCS365 (Fig. 4D). We also observed that WCS365 mutants that maintained protective ability, such as WCS365 Δ*eptA*, had lower levels of both N2C3-*lacZ* and total bacteria (Fig. S8). Meanwhile, nonprotective WCS365 deletion mutants Δ*colR*, Δ*warB*, and Δ*pap2_2* had higher N2C3-*lacZ* loads compared to plants coinoculated with the wild type WCS365 (Fig. S8). These data support the observation among diverse Pseudomonas strains that protection correlates with rhizosphere colonization. Furthermore, only a subset of ColR-dependent genes, which have rhizosphere fitness defects, were found to be necessary for protection.

### Commensal Pseudomonas can protect against an agricultural pathogen.

**FIG 5:**
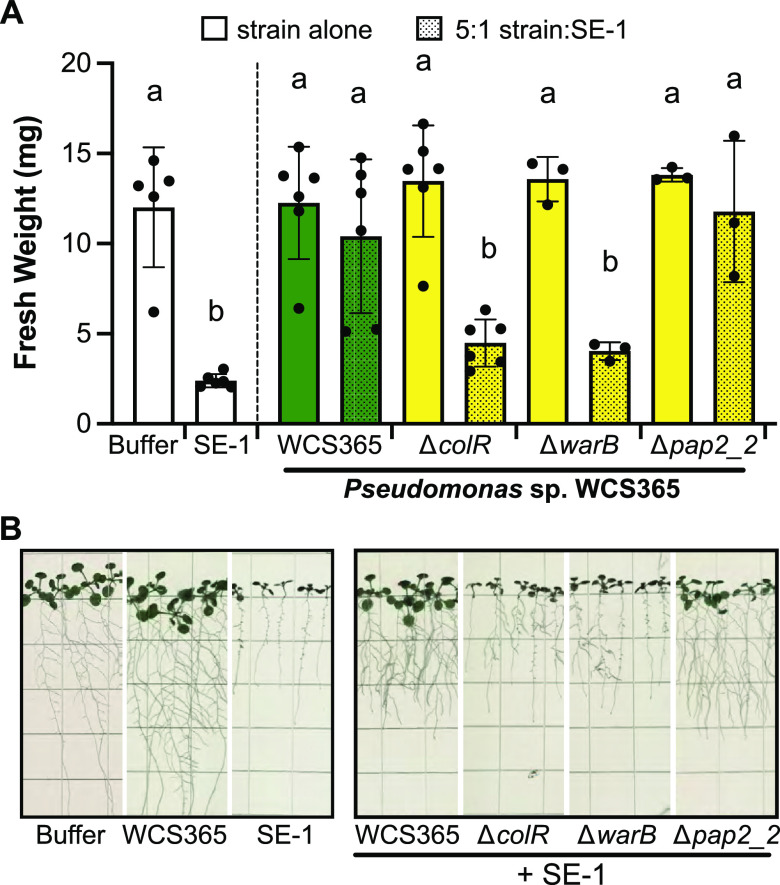
WCS365 protects against the agronomically important Pseudomonas fuscovaginae pathogen SE-1 in a *colR-* and *warB*-dependent manner. (A) WCS365 mutants with deletions in *colR* or *warB* are unable to protect against SE-1, the causal agent of bacterial sheath brown rot of rice, when coinoculated onto *Arabidopsis* roots at a 5:1 ratio. Letters indicate *P* < 0.05 determined by one-way ANOVA and Tukey HSD. (B) Representative images showing the data quantified in (A).

To determine how broadly relevant the system we developed is to identify members of the microbiome that can protect against Pseudomonas pathogens, we tested whether Pseudomonas sp. WCS365 was able to protect against *P. fuscovaginae* strain SE-1. SE-1 is the causal agent of rice brown sheath rot, and it uses syringotoxin and fuscopeptin, which are cyclic lipopeptides similar to syringomycin and syringopeptin, as virulence factors (35, 36). We found that WCS365 is able to protect against *P. fuscovaginae* strains SE-1, in a *colR-* and *warB*-dependent manner (Fig. 5). Meanwhile, *pap2_2* was not necessary for protection against SE-1. Interestingly, although WCS365 rescued fresh weight, shoot growth, and lateral root formation in the presence of SE-1, it did not fully rescue primary root stunting. Collectively these data indicate that the described gnotobiotic assay may be able to broadly identify commensal strains and mechanisms that protect from agriculturally important pathogens.

## DISCUSSION

Using a reductionist system consisting of *Arabidopsis*, an opportunistic Pseudomonas pathogen, and commensal Pseudomonas, we identified strains and genes required for members of the rhizosphere microbiome to protect against pathogens. We found that the ability to protect against pathogenesis by Pseudomonas sp. N2C3 was almost uniquely present within the BCM clade, indicating that strains closely related to N2C3 could protect against its pathogenesis, while more distantly related Pseudomonas strains could not (Fig. 1A). However, unlike many traits that show phylogenetic signatures within the genus Pseudomonas (16, 17, 23, 37), we were unable to identify components of the Pseudomonas accessory genome that correlate with protection. As a result, this work implicates variation in regulation of components of the core genome in protection against an opportunistic pathogen.

A subset of previously identified ColR-dependent genes, *colR*, *warB*, and *pap2_2* were found to be necessary for protection against N2C3 by WCS365 (Fig. 4B). However, among these genes, *pap2_2* was not necessary for protection against SE-1 (Fig. 5). This suggests that a ColR-dependent mechanism may be broadly required for outcompeting pathogens, but that specific components of the ColR regulon required for protection may vary between pathogens.

ColR has been previously reported to impact rhizosphere colonization by affecting membrane permeability via WarB (38). *Pap2_2* is predicted to encode a phosphatidic acid phosphatase, which may also impact the cell membrane by regulating phospholipid recycling and homeostasis (32). As ColR has been identified in multiple screens for rhizosphere fitness determinants, this may indicate that the Δ*colR* mutant has the most severe colonization/fitness defect of those tested (28, 29, 39). However, as not all colonization factors or *colR*-dependent genes were necessary for WCS365 to protect against N2C3 (Fig. 3C, 4B), a specific *colR*-dependent colonization mechanism, potentially involved in membrane integrity, may be required for protection.

We found that the protective strain Pseudomonas sp. WCS365 was unable to inhibit N2C3 growth *in vitro* (Fig. S7) suggesting that inhibition requires the presence of a plant. This was also supported by our discovery that ColR was necessary for protection. Since ColR is the response regulator of a two-component system, the associated sensor ColS may respond to a plant-derived signal to induce ColR phosphorylation, DNA binding, and transcriptional activation of downstream genes involved in protection. We speculate that ColR-regulated genes could contribute to higher rhizosphere colonization and pathogen protection by increasing nutrient uptake, increasing resistance to plant immune responses, or increasing production of biocontrol agents. ColR may help with these functions specifically at low pH, as ColR is required for WCS365 growth in low pH conditions such as the rhizosphere (31, 40).

Protective strains coinoculated with N2C3 tend to maintain approximately the same coinoculation ratio 7 days after inoculation (Fig. 3A, 4C). This suggests that N2C3 is not excluded by the protective strains, but rather persists as a commensal member of the microbiome. This suggests that N2C3 transitions between pathogenic and nonpathogenic lifestyles, depending on the presence of specific microbiota, which is reminiscent of opportunistic pathogens (41). Closely related Pseudomonas strains with markers of both commensal and pathogenic lifestyles (16, 20) and NFACC strains described in this study have been identified within the microbiome of the same plant suggesting competition may be ecologically relevant. We speculate that protective strains, such as the closely related BCM strains, have a similar metabolic potential as N2C3 and therefore if they colonize at high enough levels, they can restrict pathogen growth and suppress disease. This also suggests that lack of a species rich and functionally diverse community of microbiota in the soil could lead to increased opportunities for pathogens to invade as there would be less likelihood of niche overlap and competition with the pathogen (42–44).

As we found that Pseudomonas sp. WCS365 was also able to protect against *P. fuscovaginae* SE-1, our gnotobiotic system may be able to be used for identifying strains and mechanisms to control a wide range of agriculturally important pathogens. Further research on communities of beneficial bacteria could play a vital role in harnessing the genetic potential of the plant microbiome, and prevent future crop loss due to pathogen infection.

## MATERIALS AND METHODS

### Bacterial cultures and growth media.

Strains and mutants used in this work are summarized in Tables S1A and S1B, respectively. All Pseudomonas spp. were routinely cultured on King’s B or LB agar plates, and incubated at 28°C. Escherichia coli and Chromobacterium violaceum CV026 were cultured on LB agar plates, and incubated at 37°C and 28°C, respectively. Overnight cultures of Pseudomonas spp. and C. violaceum CV026 were prepared in 5 ml of LB, grown at 28°C, and shaken at 180 rpm. Overnight cultures of E. coli were prepared in 5 ml of LB, grown at 37°C, and shaken at 180 rpm. When required, growth medium was supplemented with 25 μg/ml gentamicin, 10 μg/ml nalidixic acid, 10% sucrose, or 0.2 mg/ml X-gal.

NFACC and NFIX strains used in this study are a part of a bacterial collection isolated from the roots of Switchgrass (*Panicum virgatum*) growing in Tallgrass Prairie Preserve, OK, USA. Approximately 10 pieces of healthy roots (1 cm long) were surface sterilized and crushed with a pestle to extract sap. Diluted sap were plated on 869 media. After 3–4 days of incubation, 3–4 colonies of different shapes were purely isolated from each plant. Their genomes are available at https://genome.jgi.doe.gov/portal/Comgentwoseasons/Comgentwoseasons.info.html.

### Plant growth conditions.

Arabidopsis thaliana Col-0 seeds were sterilized by submersion in 70% ethanol for 2–3 min, then 10% bleach for 1–2 min. The seeds were rinsed three times using sterile deionized water, then submerged into 0.1% phytoagar. The seeds were stored in the dark at 4°C for at least 2 days prior to sowing. The plants were grown in a growth room or a growth chamber at 22°C, under a 16h light/8h dark cycle, under 100 μM fluorescent white light and ambient humidity.

### Axenic root inoculation assays.

Sterilized seeds were planted onto square plates containing 0.5X Murashige and Skoog (MS) media, with 0.5 g/liter 2-(N-morpholino)ethanesulfonic acid (MES) buffer, 2% sucrose, and 1% phytoagar. The plates were sealed using Micropore tape and placed upright, allowing seedlings to germinate along the surface of the media. After 6 days, the seedlings were carefully transferred onto square plates containing 0.5X MS media, with 0.5 g/liter MES buffer, no sucrose, and 1% phytoagar, before being resealed and returned to the growth room. On day 7, seedlings were inoculated along their roots with 5 μl of bacterial treatments, prepared as described below. 5 seedlings were inoculated per treatment.

Bacteria were prepared by inoculating single colonies into 5 ml of LB, and grown with shaking overnight. The next day, 1 ml of overnight culture was centrifuged for 1 min at 10000 rcf, and the pelleted cells were resuspended in 1 ml of 10 mM MgSO_4_. The resuspended cells were serially diluted to an approximate OD_600_ of 0.001 using 10 mM MgSO_4_. Bacterial mixtures were prepared at 5:1 ratios of test strain:N2C3 where “1” is 10 μl and “5” is 50 μl. For example, a 5:1 treatment of WCS365:N2C3 would contain 50 μl of WCS365 and 10 μl of N2C3.

After bacterial inoculation, the plates were resealed and the plants were grown vertically for 7 days in the growth room. Images of the plates were then scanned, and seedlings from the same treatment were pooled for fresh weight measurements.

### Rhizosphere CFU counts.

N2C3-*lacZ* and WCS365-*lacZ* strains were generated through biparental mating of N2C3 or WCS365 with E. coli WM3064 or CC18 containing *pMini-Tn5-lacZ*, respectively. The relative fitness ratio (⍵) of *lacZ* strains was calculated for the transconjugant versus the parental strain *in planta* from 7–14 days *in planta* (45). For N2C3-*lacZ* versus N2C3 ⍵ = 0.993 ± 0.024 and for WCS365-*lacZ* versus WCS365 ⍵ = 1.012 ± 0.038 indicating that the LacZ transposon insertion did not result in a significant change in fitness in either strain. For quantification of whole-seedling CFU (CFU) counts, either N2C3 or WCS365 strains containing a *pMini-Tn5-lacZ* insertion were used to coinoculate 7-day-old *Arabidopsis* seedlings, as described in the Axenic Root Inoculation Assay protocol.

After 7 days, whole seedlings were pooled and sterilely transferred into a 2 ml microcentrifuge tube containing a metal bead and 500 μl of 10 mM MgSO_4_. Fresh weights were measured for each treatment. Plant cells were homogenized using a Qiagen TissueLyser II at 30 Hz for 2 min. Tissue lysate was serially diluted and plated onto LB agar supplemented with 0.2 mg/ml 5-Bromo-4-Chloro-3-Indolyl β-d-Galactopyranoside (X-gal). Plates were incubated at 28°C for 2 days. Blue and white colonies were counted, to calculate the ratio of blue LacZ-containing cells and white coinoculated cells in the rhizosphere. Absolute CFU were calculated per mg of plant tissue.

### AHL biosensor assays.

Overnight cultures of Pseudomonas sp. WCS365, Pseudomonas sp. N2C3, and C. violaceum CV026 were resuspended then diluted in 10 mM MgSO_4_, to an OD_600nm_ of 2. Bacterial treatments were prepared by mixing 5:1 ratios of WCS365:N2C3. Using an inoculation loop, bacterial treatments were streaked along an LB agar plate. CV026 was streaked beside each bacterial treatment. Plates were incubated at 28°C and imaged after 2 days and production of violacein by CV026 was recorded.

### Comparative genomics using PyParanoid.

Comparative genomics was performed using the PyParanoid pipeline described previously (16, 17). Briefly, the PyParanoid pipeline was previously used to create a database using 3886 Pseudomonas genomes, identifying 24066 homologous protein families (“gene groups”) that covered 94.2% of the generated Pseudomonas pangenome. The presence or absence of gene groups were compared in strains with protection phenotypes determined via axenic root inoculation assays.

### Phylogenetic trees.

Phylogenetic trees were generated using the PyParanoid pipeline and FastTree 2 as described previously (16, 17). Briefly, an alignment of 122 single-copy genes conserved within the Pseudomonas genus was created previously. FastTree 2 was used to generate a phylogenetic tree from this alignment, by randomly sampling 5000 amino acid residues without replacement. Strains were then subset from this alignment to create phylogenetic trees, using FastTree 2.

### Generating gene deletions.

Gene deletions in Pseudomonas sp. WCS365 were created using a two-step allelic exchange method described previously (46). For each target gene deletion, 700–900 bp regions immediately flanking upstream and downstream of the target region were amplified using WCS365 genomic DNA as a template, and the primers listed in Table S1C. The amplified flanking regions were then joined using overlap PCR. The overlap PCR product was digested and ligated into the pEXG2 suicide vector, and transformed into competent Escherichia coli DH5α cells via heat shock. Correct insertions were confirmed by colony PCR and Sanger sequencing, before transforming the plasmid into E. coli SM10λpir. Deletion plasmids were conjugated into WCS365 through biparental mating. Transconjugants that underwent a single-crossover event allowing for site-specific chromosomal integration were selected for using LB agar supplemented with 25 μg/ml of gentamicin and 15 μg/ml of nalidixic acid. After re-streaking and validating antibiotic resistance, transconjugants were grown overnight in no-salt LB at 37°C, and cells that underwent a double crossover to excise the plasmid backbone were selected by plating on LB agar supplemented with 10% sucrose. Successful gene deletions were confirmed using PCR, gel electrophoresis, and Sanger sequencing. The Pseudomonas sp. WCS365 genome is available as accession CP089973 (31).

### Cross-streak assays.

Bacteria were grown overnight in LB, at 28°C. Using a sterile inoculation loop, the first bacterial strain was streaked vertically onto LB plates, directly from the overnight culture. The plate was allowed to dry before streaking the second strain perpendicular to the first. The plate was incubated overnight at 28°C.

### Statistical analyses.

All statistical analyses were performed using Prism 9 software. In general, competition assays were analyzed by one-way ANOVA and Tukey’s HSD test and LacZ assays were analyzed using two-way ANOVA and Tukey’s HSD test.
