# Supplementary figures and images for: Commensal Pseudomonas fluorescens Strains Protect Arabidopsis from Closely Related Pseudomonas Pathogens in a Colonization-Dependent Manner
Source: mBio. 2022 Feb 1;13(1):e02892-21. doi: 10.1128/mbio.02892-21 (PMC8805031; doi:10.1128/mbio.02892-21)

**A**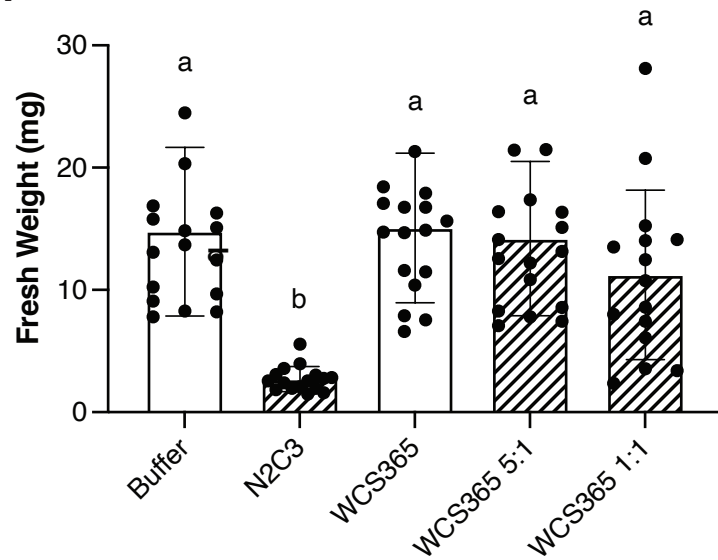**B**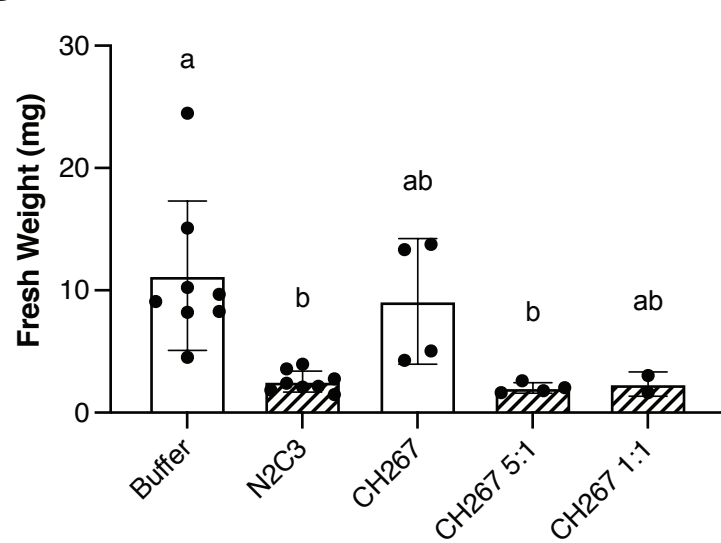**C****WCS365**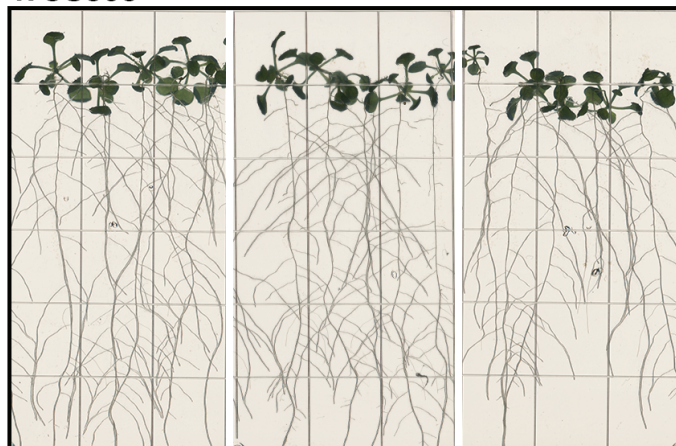

Alone

5:1

1:1

**CH267**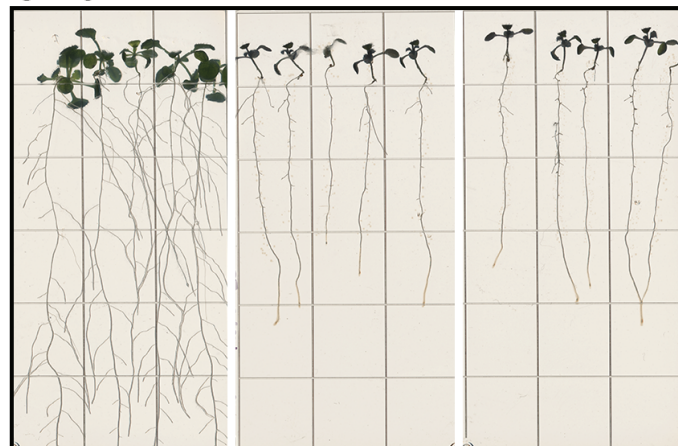

Alone

5:1

1:1

Supplement: FIG S1 [file mbio.02892-21-sf001.pdf]

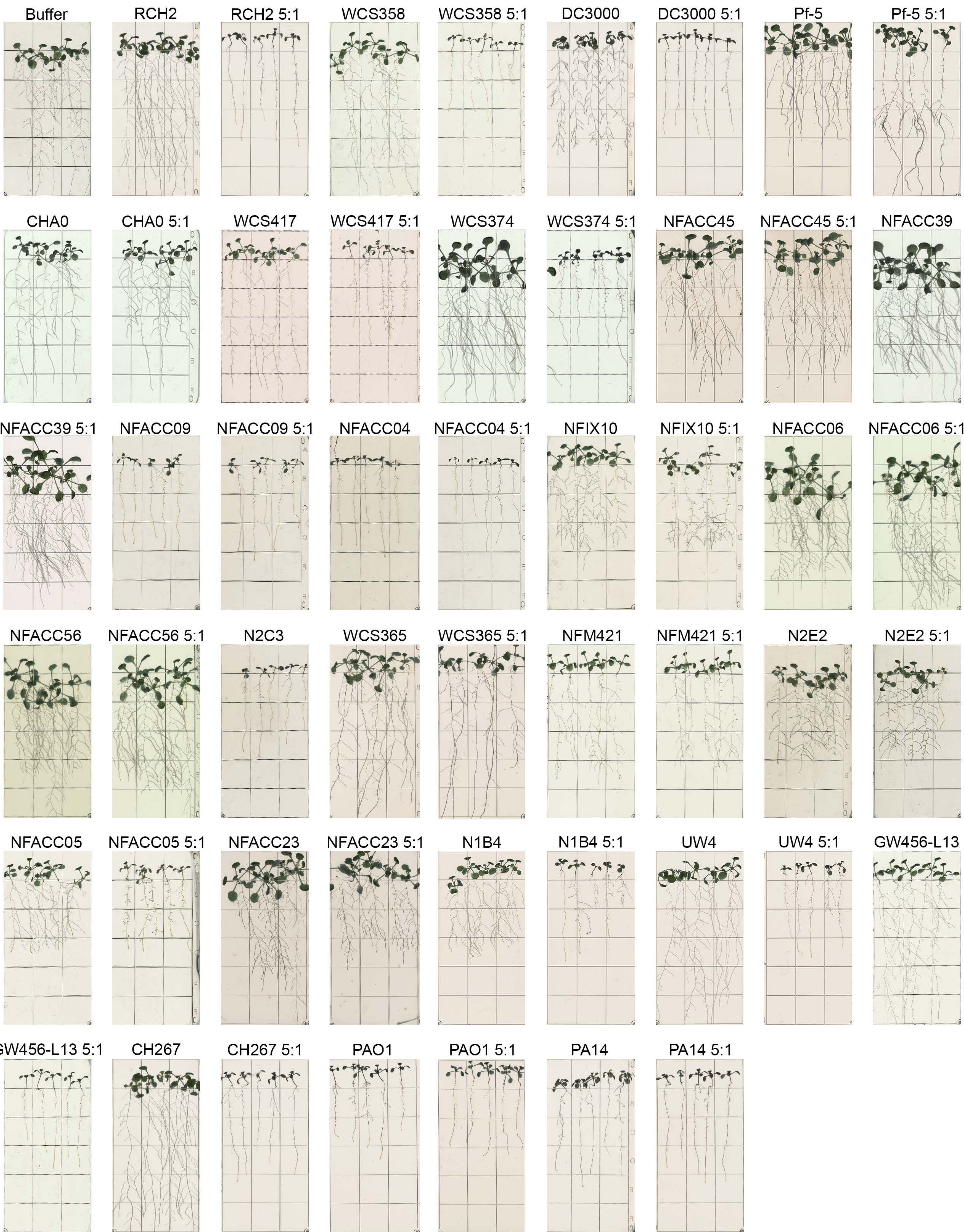

Supplement: FIG S2 [file mbio.02892-21-sf002.pdf]

**A**

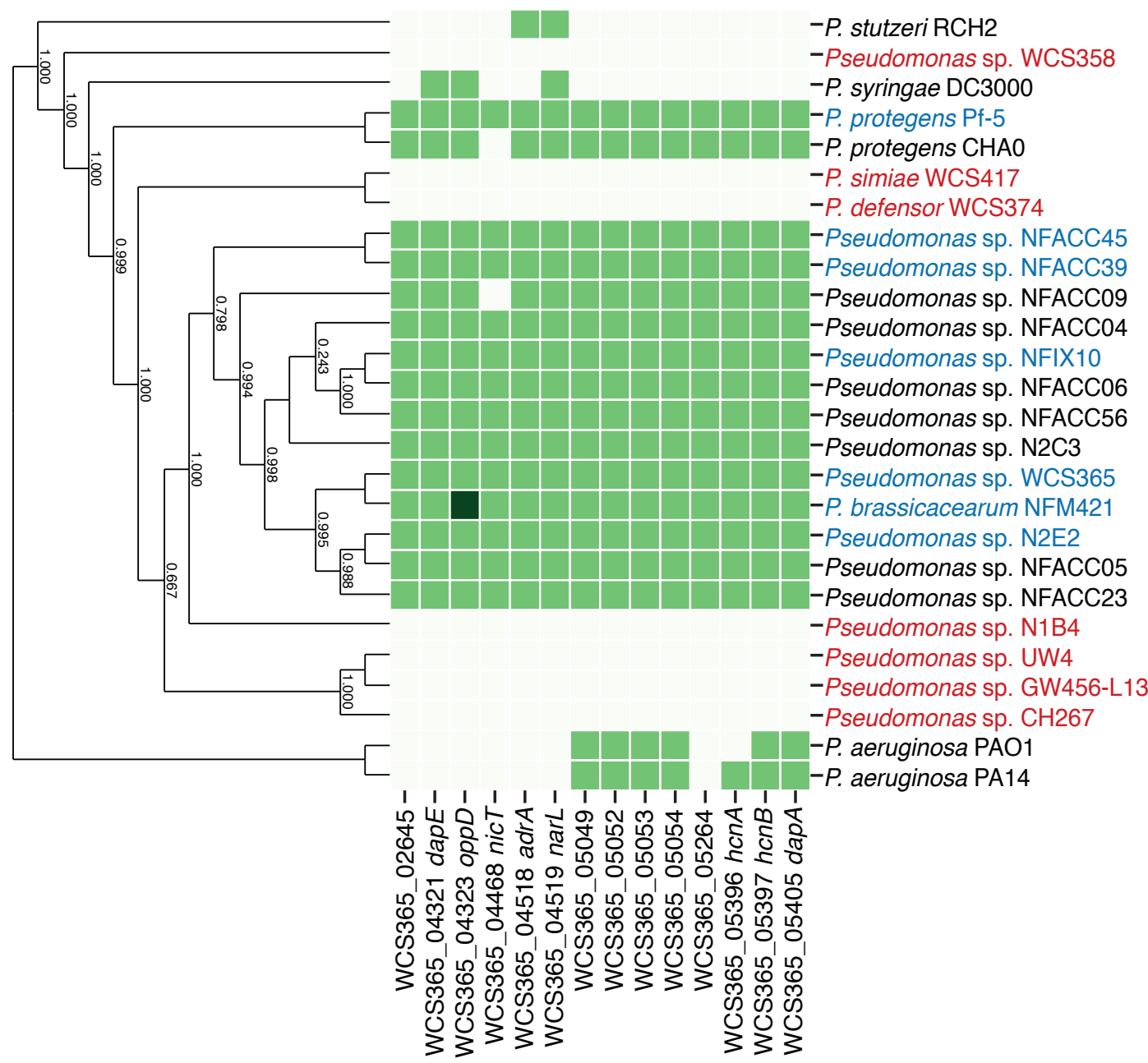

**B**

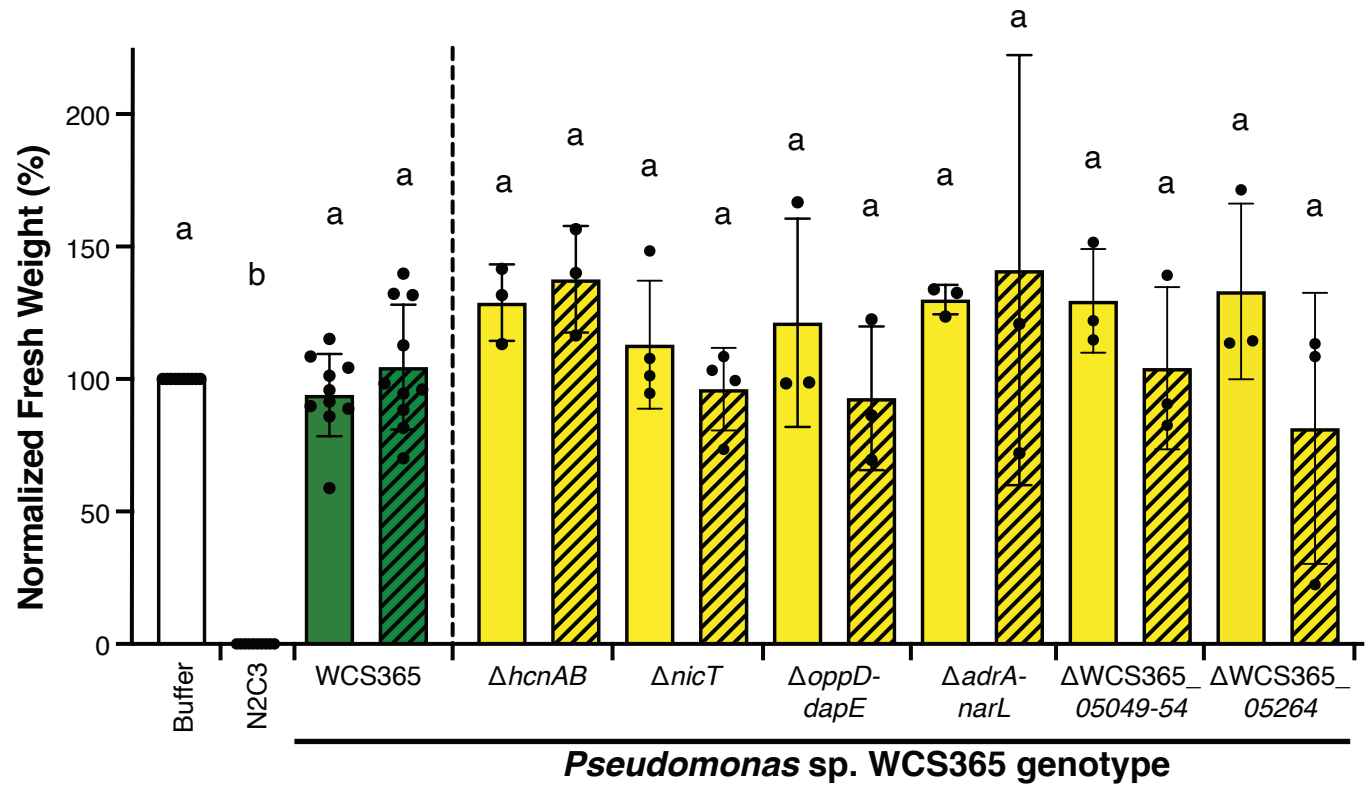

Supplement: FIG S3 [file mbio.02892-21-sf003.pdf]

**A**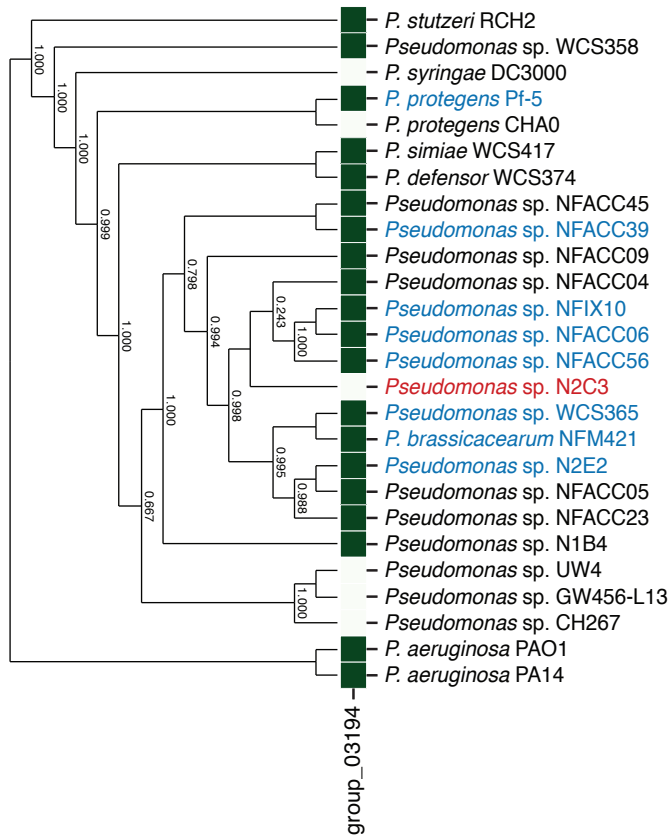**B**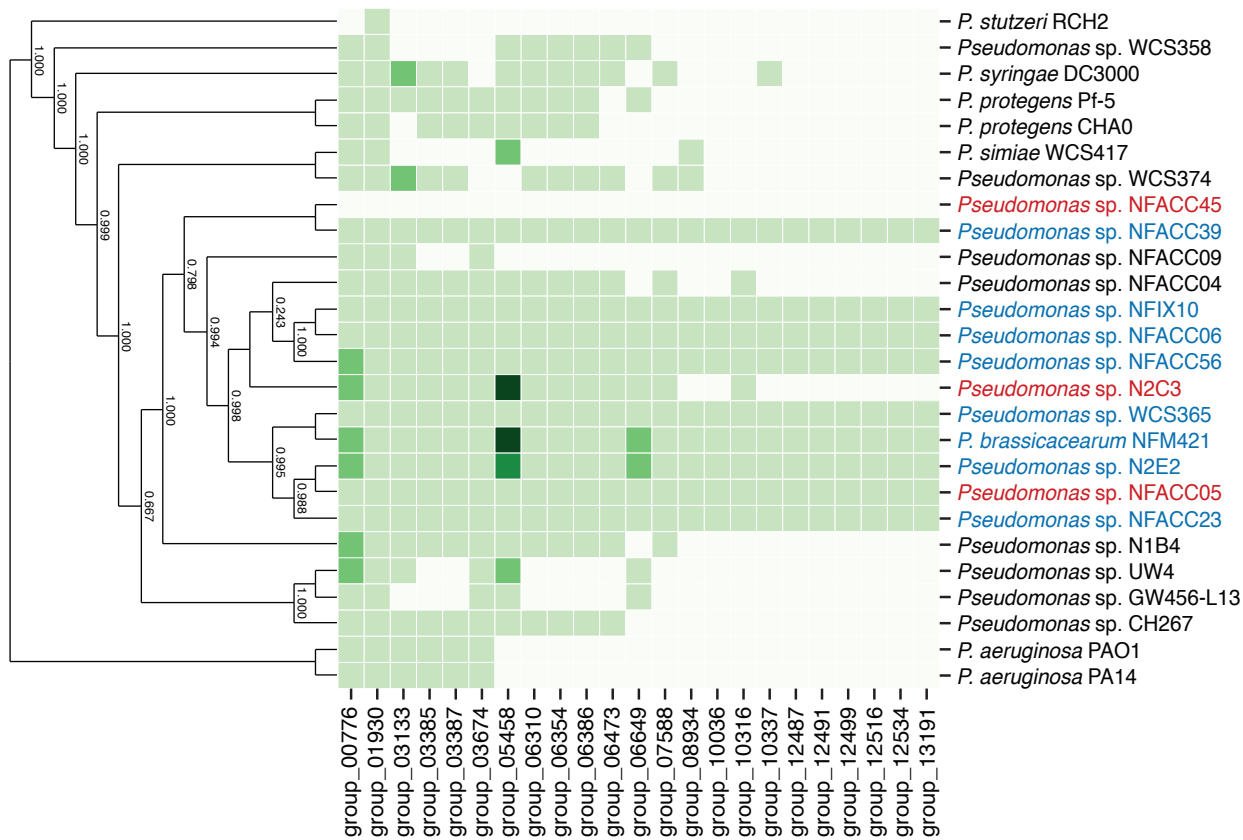

Supplement: FIG S4 [file mbio.02892-21-sf004.pdf]

**A**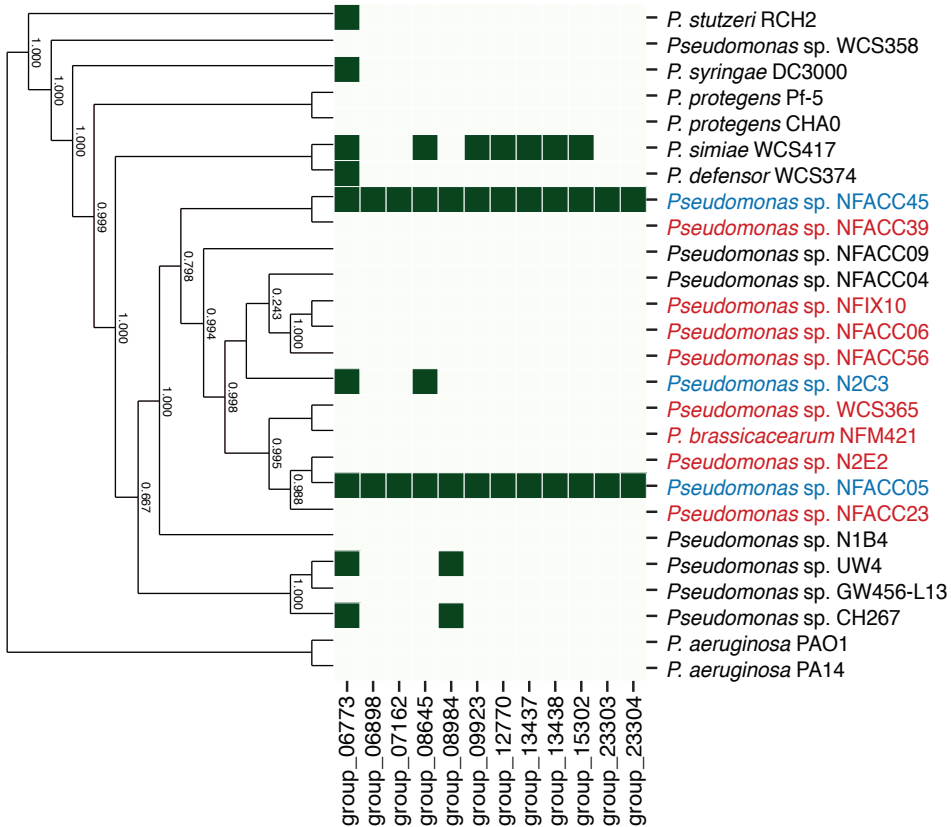**B**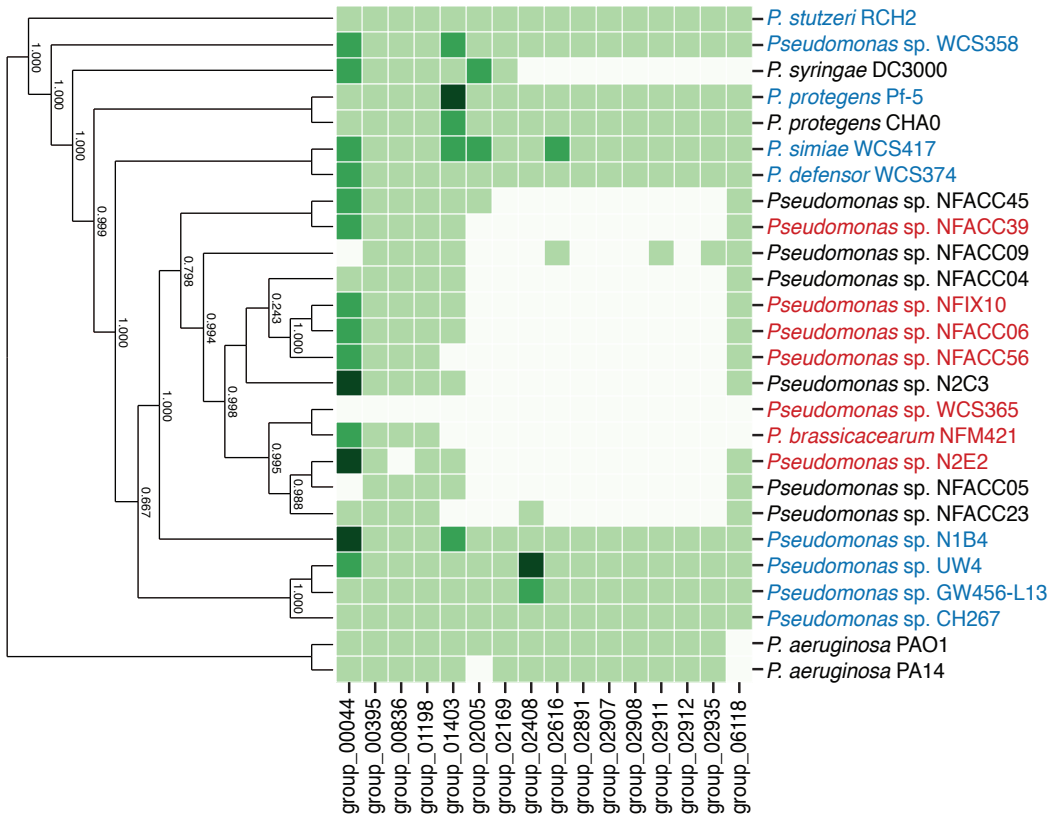

Supplement: FIG S5 [file mbio.02892-21-sf005.pdf]

**A**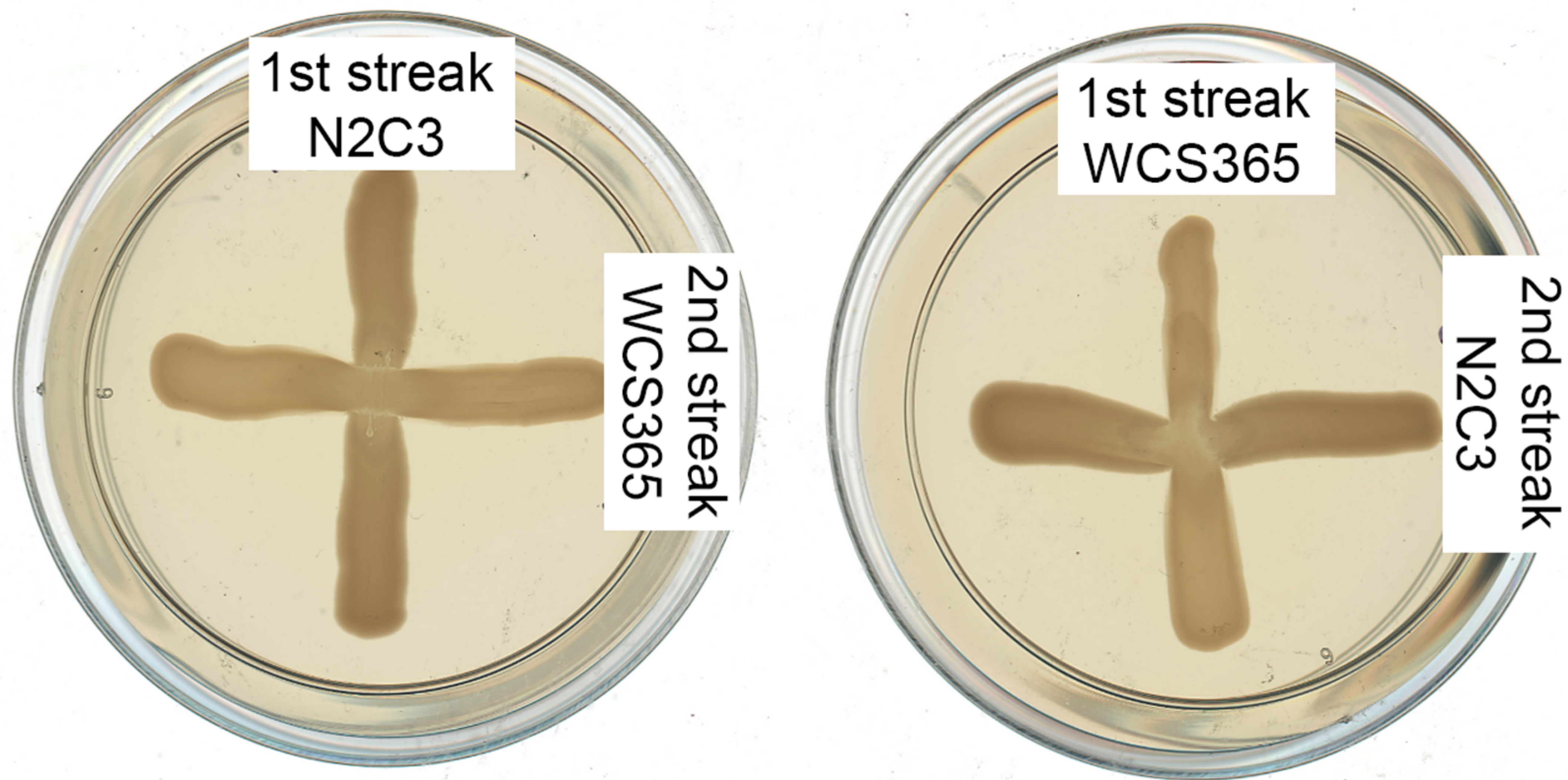**B**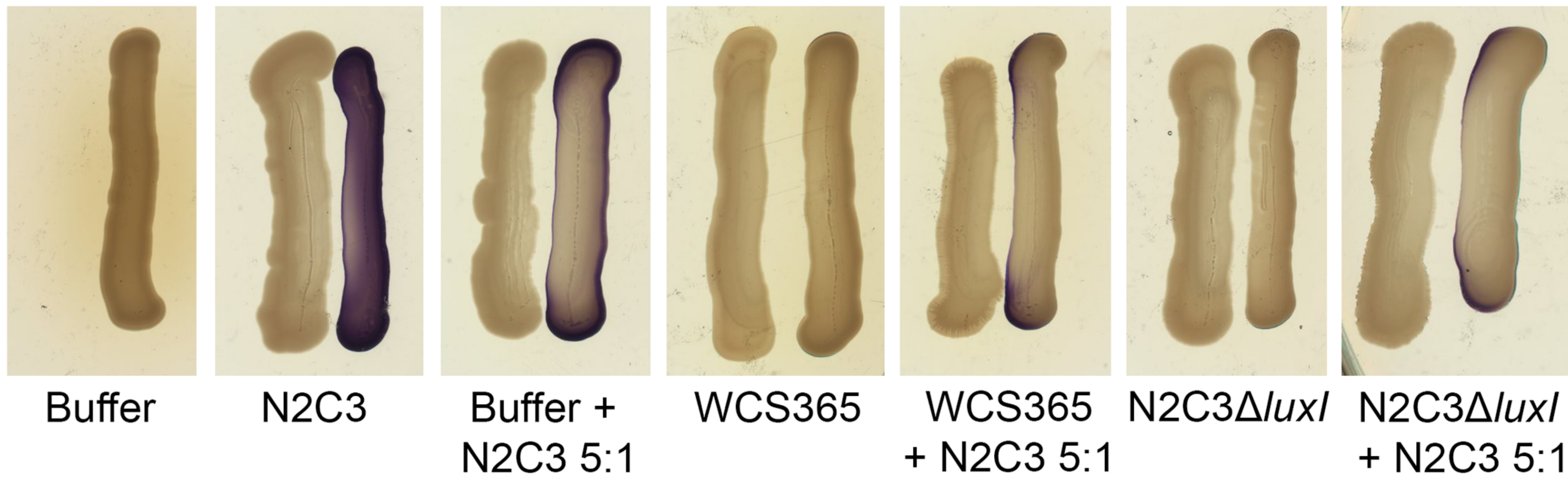

Supplement: FIG S7 [file mbio.02892-21-sf007.pdf]

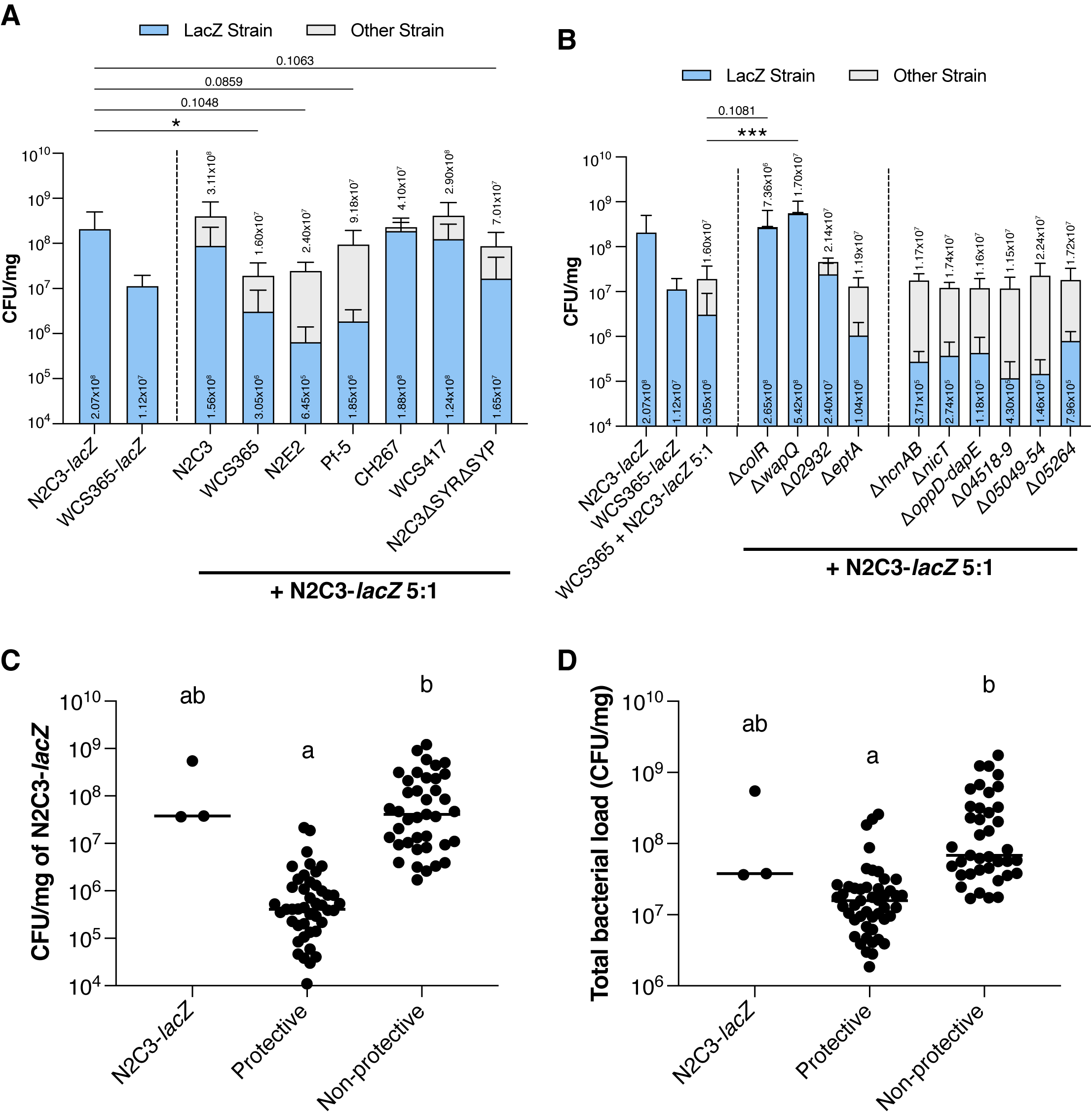

Supplement: FIG S8 [file mbio.02892-21-sf008.tif]

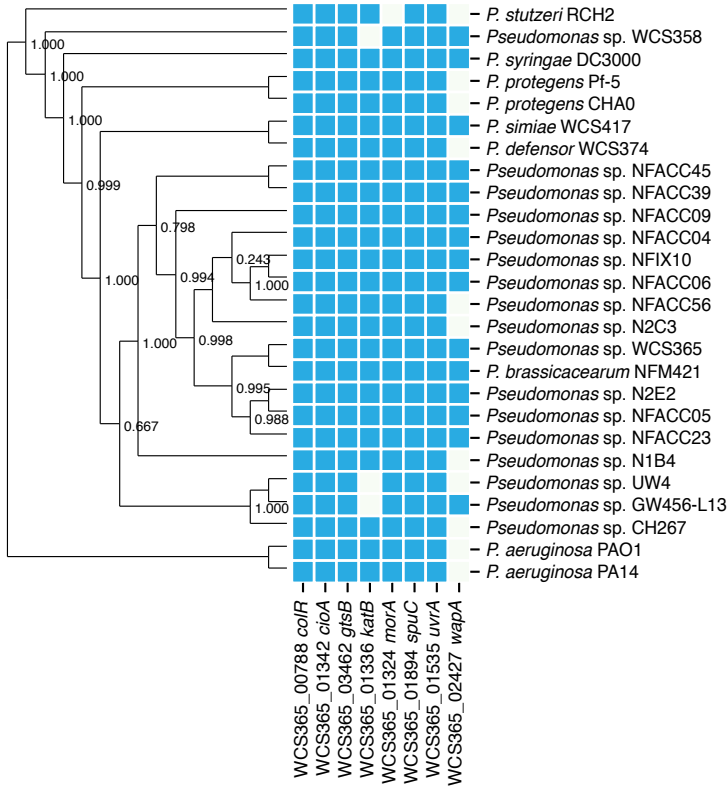

Supplement: FIG S9 [file mbio.02892-21-sf009.pdf]
