# Supplementary material for: Commensal Pseudomonas fluorescens Strains Protect Arabidopsis from Closely Related Pseudomonas Pathogens in a Colonization-Dependent Manner
Source: mBio. 2022 Feb 1;13(1):e02892-21. doi: 10.1128/mbio.02892-21 (PMC8805031; doi:10.1128/mbio.02892-21)
Supplement: TABLE S1 [file mbio.02892-21-st001.docx]

**Table S1. Strains (A), mutants (B), and primers (C) used in this study**

**(A)**

| **Strain Name** | **Source** | **Location** | **Reference** |
| --- | --- | --- | --- |
| *P. protegens* CHA0 | Tobacco rhizosphere | Switzerland | (1) |
| *Pseudomonas* sp. CH267 | *Arabidopsis* rhizosphere | Cambridge, MA, USA | (2) |
| *P. syringae* DC3000 | Tomato leaves | Guernsey, UK | (3, 4) |
| *Pseudomonas* sp. FW300-N1B4 | Groundwater | Oak Ridge, TN, USA | (5) |
| *Pseudomonas* sp. FW300-N2C3 | Groundwater | Oak Ridge, TN, USA | (5) |
| *Pseudomonas* sp. FW300-N2E2 | Groundwater | Oak Ridge, TN, USA | (6) |
| *Pseudomonas* sp. GW456-L13 | Groundwater | Oak Ridge, TN, USA | (5) |
| *Pseudomonas* sp. NFACC04 | Switchgrass rhizosphere | Osage County, OK, USA | This study |
| *Pseudomonas* sp. NFACC05 | Switchgrass rhizosphere | Osage County, OK, USA | This study |
| *Pseudomonas* sp. NFACC06 | Switchgrass rhizosphere | Osage County, OK, USA | This study |
| *Pseudomonas* sp. NFACC09 | Switchgrass rhizosphere | Osage County, OK, USA | This study |
| *Pseudomonas* sp. NFACC23 | Switchgrass rhizosphere | Osage County, OK, USA | This study |
| *Pseudomonas* sp. NFACC39 | Switchgrass rhizosphere | Osage County, OK, USA | This study |
| *Pseudomonas* sp. NFACC45 | Switchgrass rhizosphere | Osage County, OK, USA | This study |
| *Pseudomonas* sp. NFACC56 | Switchgrass rhizosphere | Osage County, OK, USA | This study |
| *Pseudomonas* sp. NFIX10 | Switchgrass rhizosphere | Osage County, OK, USA | This study |
| *P. brassicacearum* NFM421 | *Arabidopsis* rhizoplane | Méréville, France | (7) |
| *P. aeruginosa* PA14 | Human wound | Boston, MA, USA | (8) |
| *P. aeruginosa* PAO1 | Human wound | Melbourne, Australia | (9) |
| *P. protegens* Pf-5 | Cotton rhizosphere | College Station, TX, USA | (10) |
| *P. stutzeri* RCH2 | Groundwater | Hanford, WA, USA | (11) |
| *P. fuscovaginae* SE-1 | *Oryza sativa* | Siniloan, Philippines | (12) |
| *Pseudomonas* sp. UW4 | Reed rhizosphere | Waterloo, ON, Canada | (13) |
| *Pseudomonas* sp. WCS358 | Potato rhizosphere | Netherlands | (14, 15) |
| *Pseudomonas* sp. WCS365 | Potato rhizosphere | Netherlands | (14) |
| *P. defensor* WCS374 | Potato rhizosphere | Netherlands | (14, 15) |
| *P. simiae* WCS417 | Wheat rhizosphere | Netherlands | (16) |

**(B)**

| **Mutant Name** | **Locus Tag** | **Predicted Function** | **Reference** |
| --- | --- | --- | --- |
| N2C3 ΔSYRΔSYP |  | Syringomycin and syringopeptin production | (17) |
| WCS365 Δ*hrcC* | WCS365_01864 | Type III secretion system component | This study |
| WCS365 ΔDAPG | WCS365_04453-04458 | 2,4-Diacetylphloroglucinol | This study |
| WCS365 Δ*colR* | WCS365_00788 | Two component system response regulator | (18) |
| WCS365 Δ*cioA* | WCS365_01342 | Cytochrome oxidase subunit | (19) |
| WCS365 Δ*gtsB* | WCS365_03452 | Glucose ABC transporter permease | (19) |
| WCS365 Δ*katB* | WCS365_01336 | Catalase | (19) |
| WCS365 Δ*morA* | WCS365_01324 | Cyclic di-GMP phosphodiesterase | (19) |
| WCS365 Δ*spuC* | WCS365_01894 | Putrescine aminotransferase | (19) |
| WCS365 Δ*uvrA* | WCS365_01535 | Excinuclease | (19) |
| WCS365 Δ*wapA* | WCS365_02427 | Nuclease | (19) |
| WCS365 Δ*warA* (also known as Δ*orf222)* | WCS365_00786 | Methyltransferase | This study, (18) |
| WCS365 Δ*warB* (also known as Δ*wapQ*) | WCS365_00785 | Heptose kinase | This study, (18) |
| WCS365 Δ*tpbA* | WCS365_02280 | Tyrosine phosphatase | This study, (20) |
| WCS365 Δ*pap2_2* | WCS365_02932 | Phosphatidic acid phosphatase | This study, (21) |
| WCS365 Δ*eptA* | WCS365_03626 | Phosphoethanolamine transferase | (22) |
| WCS365 Δ*catBCA* | WCS365_05038-WCS365_05040 | Catalase production | This study (23) |
| WCS365 Δ*02645* | WCS365_02645 | Soluble epoxide hydrolase | This study |
| WCS365 Δ*oppD-dapE* | WCS365_04321-WCS365_04323 | oppD- Oligopeptide transport ATP-binding protein  dapE- Succinyl-diaminopimelate desuccinylase | This study |
| WCS365 Δ*nicT* | WCS365_04468 | nicT- Metabolite transport protein | This study |
| WCS365 Δ*ardA-narL* | WCS365_04518-WCS365_04519 | ardA- Diguanylate cyclase  narL- Transcriptional regulatory protein | This study |
| WCS365 Δ*05049-54* | WCS365_05049-WCS365_05054 | Unknown function | This study |
| WCS365 Δ*hcnAB* | WCS365_05396-WCS365_05397 | Hydrogen cyanide production | This study |
| WCS365 Δ*dapA* | WCS365_05405 | 4-hydroxy-tetrahydrodipicolinate synthase | This study |
| N2C3 Δ*luxI* | AO356_RS28195 | AHL synthase | (17) |

(C)

| **Deletion Strain** | **Primer Type** | **Primer Sequence (5' ⟶ 3')** | **RE Site** |
| --- | --- | --- | --- |
| WCS365 Δ*hrcC* | Upstream forward | GNNAAGCTTTGCCTGTGAACACCCCGGAA | HindIII |
|  | Upstream reverse | ACCATCGACTGCATTTATATAGTGTACTTTGCCTGCGGTCAT |  |
|  | Downstream forward | TATATAAATGCAGTCGATGGTGTCATGAAACAACCCACGCG |  |
|  | Downstream reverse | GNNAAGCTTGAGCTATGGAGGATGGCGAC | HindIII |
| WCS365 ΔDAPG | Upstream forward | GNNAAGCTTGCAATTCACGCGACGTATCC | HindIII |
|  | Upstream reverse | ACCATCGACTGCATTTATATACGCGCCATAGCTCACAATTC |  |
|  | Downstream forward | TATATAAATGCAGTCGATGGTGGCTGTATTGACCGGCCCCTG |  |
|  | Downstream reverse | GNNAAGCTTGACCAGTGAGAGTGTCGAGC | HindIII |
| WCS365  Δ*eptA* | Upstream forward | ATATGAATTCGCATGCACATGCATGTCAGCC | EcoRI |
|  | Upstream reverse | GACAGGTTAAACAACGCTGCGTGACGGCTTCCTGAAAGTTGCTC |  |
|  | Downstream forward | GAGCAACTTTCAGGAAGCCGTCAACGCAGCGTTGTTTAACCTGTC |  |
|  | Downstream reverse | ATATGGATCCGCCTAAGAGAAATCGCCTGCG | BamHI |
|  | Upstream confirmation | GCAGGCCTGCTTCATGGC |  |
|  | Downstream confirmation | CTTGAAGAATGGCGCGCAG |  |
| WCS365  Δ*warB* | Upstream forward | ATATGAATTCCCCATCAATGCCCGACCC | EcoRI |
|  | Upstream reverse | CTGCCAGGGCTCCGACTAAAATCGTTGATGGTTCTGCTGCC |  |
|  | Downstream forward | AATCGTTGATGGTTCTGCTGCCTTTAGTCGGAGCCCTGGCAG |  |
|  | Downstream reverse | ATATGGATCCGCGTCGACTCGTCTGC | BamHI |
|  | Upstream confirmation | ACGGTTGCCGTTTCGAGG |  |
|  | Downstream confirmation | GCCGTGGAACAAGCCGC |  |
| WCS365  Δ*warA* | Upstream forward | ATATGAATTCTTGGCGACCTCCTGCG | EcoRI |
|  | Upstream reverse | CCTGAATACGAAACCCTGCCTGTGATGGGGGCCTCCATGTC |  |
|  | Downstream forward | GACATGGAGGCCCCCATCACAGGCAGGGTTTCGTATTCAGG |  |
|  | Downstream reverse | ATATGGATCCCGACGAGTGGCGCCG | BamHI |
|  | Upstream confirmation | CCGTGGCGTTCGACGC |  |
|  | Downstream confirmation | AACTGCCAGGGCTCCG |  |
| WCS365  Δ*pap2_2* | Upstream forward | ATATGAATTCCCTCTCTACTGTCGCGCCAAC | EcoRI |
|  | Upstream reverse | CGTGATTGGGTACAGTCGCCTCAGATTGAGCCCGTAGAAACGGG |  |
|  | Downstream forward | CCCGTTTCTACGGGCTCAATCTGAGGCGACTGTACCCAATCACG |  |
|  | Downstream reverse | ATATGGATCCCGAGTTCAACGCAGCCCAATC | BamHI |
|  | Upstream confirmation | TCTGCCACAGAATCGAGGTGTC |  |
|  | Downstream confirmation | CCTTCCGTGGTTCCGACAAAC |  |
| WCS365  Δ*catBCA* | Upstream forward | ATATGAATTCCTCACGACGATGGGTGAAGTGG | EcoRI |
|  | Upstream reverse | GTTGTTCGCTCAAGACCAGGCAGTCGCCAGATCCAGCAACTGG |  |
|  | Downstream forward | CAGTTGCTGGATCTGGCGACTGCCTGGTCTTGAGCGAACAAC |  |
|  | Downstream reverse | ATATGGATCCGGCGCTCGAACAGCGTATAC | BamHI |
|  | Upstream confirmation | GCATTGTTGCAACACTGGCG |  |
|  | Downstream confirmation | GCTGTCGCGGTTTGCAGTTC |  |
| WCS365  Δ*nicT* | Upstream forward | CATCAAGCTTCAGCAGCAGCGCTATGACTT | HindIII |
|  | Upstream reverse | AGGATCAGTCCACCGCTGACGCAACTGCTCGGAGATGGTT |  |
|  | Downstream forward | AACCATCTCCGAGCAGTTGCGTCAGCGGTGGACTGATCCT |  |
|  | Downstream reverse | CATCGGATCCAATTGCCGGTAGACCTGTGC | BamHI |
|  | Upstream confirmation | ATCCTGCAGATCAAGCAGCG |  |
|  | Downstream confirmation | TGCGCGATCTCTTCAGTGTG |  |
| WCS365  Δ*hcnAB* | Upstream forward | CATCAAGCTTGCGAAGCCTGCGAACTGATC | HindIII |
|  | Upstream reverse | GCTATGGCGCAGTGTCAGTCAAACAAGTCAGGCATGGGCC |  |
|  | Downstream forward | GGCCCATGCCTGACTTGTTTGACTGACACTGCGCCATAGC |  |
|  | Downstream reverse | CATCGGATCCCGCCCCATCAGGGAACAAGA | BamHI |
|  | Upstream confirmation | TGATGGATTGGCCCTGTGCC |  |
|  | Downstream confirmation | GCCGATGATGTCATCGAGCT |  |
| WCS365  Δ*oppD-dapE* | Upstream forward | TCATGGATCCCCGATGTGCTCAACGCACTG | BamHI |
|  | Upstream reverse | CCCGCCTTGTCATAGCTGTTGCACCAGAATCATCAGGGCG |  |
|  | Downstream forward | CGCCCTGATGATTCTGGTGCAACAGCTATGACAAGGCGGG |  |
|  | Downstream reverse | CATGAAGCTTGTTCGCTGTGACAGGCTTCC | HindIII |
|  | Upstream confirmation | CGCGTGTTGTTCAAGCATGC |  |
|  | Downstream confirmation | ACGCTCGGTATGAACCTTGC |  |
| WCS365  Δa*drA-narL* | Upstream forward | TCATGGATCCCATCCTTGGCGATCCGGTTT | BamHI |
|  | Upstream reverse | CCAGCCACCCTCAATGTTGCCCATTGGCGTTCAATGGCAG |  |
|  | Downstream forward | CTGCCATTGAACGCCAATGGGCAACATTGAGGGTGGCTGG |  |
|  | Downstream reverse | GCATAAGCTTGAAAGTGGCTGGGACTTCAGC | HindIII |
|  | Upstream confirmation | GTCGAGCCGTCGACTGAAAC |  |
|  | Downstream confirmation | CCTCGACAGGAATCCTGGCT |  |
| WCS365  Δ05049-54 | Upstream forward | TCATGGATCCCGATTATCACTGGCCGCGTC | BamHI |
|  | Upstream reverse | AAACATCCGGATCAACGCCCATGGTGATGTTCTTGCCGCT |  |
|  | Downstream forward | AGCGGCAAGAACATCACCATGGGCGTTGATCCGGATGTTT |  |
|  | Downstream reverse | GCATAAGCTTTAGCTCGCCTCTGAAGAGGC | HindIII |
|  | Upstream confirmation | CTGGGATCGCCATGACCAGT |  |
|  | Downstream confirmation | AGCGTGATCTGGATATCGGCT |  |
| WCS365  Δ*05264* | Upstream forward | GCATAAGCTTGACAATCTTGGCGCGAGTCC | HindIII |
|  | Upstream reverse | CTAGTCCGGCGATGCTATCCCAGGACACGCCAGTCTGTTG |  |
|  | Downstream forward | CAACAGACTGGCGTGTCCTGGGATAGCATCGCCGGACTAG |  |
|  | Downstream reverse | TCATGGATCCCGGCATCGAAGTAGGCGTAG | BamHI |
|  | Upstream confirmation | CTCAATCGGATGGGCCATCAA |  |
|  | Downstream confirmation | CGACGGCTTCGTCGTTGATC |  |

**Supplementary References**

1. Stutz EW, Défago G, Kern H. 1986. Naturally occurring fluorescent pseudomonads involved in suppression of black root rot of tobacco. Phytopathology 76:181–185.

2. Haney CH, Samuel BS, Bush J, Ausubel FM. 2015. Associations with rhizosphere bacteria can confer an adaptive advantage to plants. Nat Plants 1:1–9.

3. Buell CR, Joardar V, Lindeberg M, Selengut J, Paulsen IT, Gwinn ML, Dodson RJ, Deboy RT, Durkin AS, Kolonay JF, Madupu R, Daugherty S, Brinkac L, Beanan MJ, Haft DH, Nelson WC, Davidsen T, Zafar N, Zhou L, Liu J, Yuan Q, Khouri H, Fedorova N, Tran B, Russell D, Berry K, Utterback T, Van Aken SE, Feldblyum T V., D’Ascenzo M, Deng W-L, Ramos AR, Alfano JR, Cartinhour S, Chatterjee AK, Delaney TP, Lazarowitz SG, Martin GB, Schneider DJ, Tang X, Bender CL, White O, Fraser CM, Collmer A. 2003. The complete genome sequence of the *Arabidopsis* and tomato pathogen *Pseudomonas syringae* pv. *tomato* DC3000. Proc Natl Acad Sci 100:10181–10186.

4. Cuppels DA. 1986. Generation and characterization of Tn5 insertion mutations in *Pseudomonas syringae* pv. *tomato*. Appl Environ Microbiol 51:323–327.

5. Price MN, Wetmore KM, Waters RJ, Callaghan M, Ray J, Liu H, Kuehl J V., Melnyk RA, Lamson JS, Suh Y, Carlson HK, Esquivel Z, Sadeeshkumar H, Chakraborty R, Zane GM, Rubin BE, Wall JD, Visel A, Bristow J, Blow MJ, Arkin AP, Deutschbauer AM. 2018. Mutant phenotypes for thousands of bacterial genes of unknown function. Nature 557:503–509.

6. Thorgersen MP, Lancaster WA, Vaccaro BJ, Poole FL, Rocha AM, Mehlhorn T, Pettenato A, Ray J, Waters RJ, Melnyk RA, Chakraborty R, Hazen TC, Deutschbauer AM, Arkin AP, Adams MWW. 2015. Molybdenum availability is key to nitrate removal in contaminated groundwater environments. Appl Environ Microbiol 81:4976–4983.

7. Achouak W, Sutra L, Heulin T, Meyer JM, Fromin N, Degraeve S, Christen R, Gardan L. 2000. *Pseudomonas brassicacearum* sp. nov. and *Pseudomonas thivervalensis* sp. nov., two root-associated bacteria isolated from *Brassica napus* and *Arabidopsis thaliana*. Int J Syst Evol Microbiol 50:9–18.

8. Wiehlmann L, Wagner G, Cramer N, Siebert B, Gudowius P, Morales G, Köhler T, Van Delden C, Weinel C, Slickers P, Tümmler B. 2007. Population structure of *Pseudomonas aeruginosa*. Proc Natl Acad Sci U S A 104:8101–8106.

9. Holloway BW. 1955. Genetic recombination in *Pseudomonas aeruginosa*. J Gen Microbiol 13:572–581.

10. Howell CR, Stipanovic RD. 1979. Control of *Rhizoctonia solani* on cotton seedlings with *Pseudomonas fluorescens* and with an antibiotic produced by the bacterium by the soil tube method described previously. Phytopathology 69:480–482.

11. Chakraborty R, Woo H, Dehal P, Walker R, Zemla M, Auer M, Goodwin LA, Kazakov A, Novichkov P, Arkin AP, Hazen TC. 2017. Complete genome sequence of *Pseudomonas stutzeri* strain RCH2 isolated from a hexavalent chromium [Cr(VI)] contaminated site. Stand Genomic Sci 12:1–9.

12. Cottyn B. 2003. Bacteria associated with rice seed from Philippine farmers’ fields. Universiteit Gent.

13. Glick BR, Karaturovíc DM, Newell PC. 1995. A novel procedure for rapid isolation of plant growth promoting pseudomonads. Can J Microbiol 41:533–536.

14. Geels FP, Schippers B. 1983. Selection of antagonistic fluorescent *Pseudomonas* spp. and their root colonization and persistence following treatment of seed potatoes. J Phytopathol 108:193–206.

15. Berendsen RL, van Verk MC, Stringlis IA, Zamioudis C, Tommassen J, Pieterse CMJ, Bakker PAHM. 2015. Unearthing the genomes of plant-beneficial *Pseudomonas* model strains WCS358, WCS374 and WCS417. BMC Genomics 16:1–23.

16. Lamers JG, Schippers B, Geels FP. 1988. Soil-borne diseases of wheat in the Netherlands and results of seed bacterization with pseudomonads against Gaeumannomyces graminis var. tritici, associated with disease resistance. Cereal Breed Relat to Integr Cereal Prod 134–139.

17. Melnyk RA, Hossain SS, Haney CH. 2019. Convergent gain and loss of genomic islands drive lifestyle changes in plant-associated *Pseudomonas*. ISME J 13:1575–1588.

18. De Weert S, Dekkers LC, Bitter W, Tuinman S, Wijfjes AHM, Van Boxtel R, Lugtenberg BJJ. 2006. The two-component *colR/S* system of *Pseudomonas fluorescens* WCS365 plays a role in rhizosphere competence through maintaining the structure and function of the outer membrane. FEMS Microbiol Ecol 58:205–213.

19. Liu Z, Beskrovnaya P, Melnyk RA, Hossain SS, Khorasani S, O’Sullivan LR, Wiesmann CL, Bush J, Richard JD, Haney CH. 2018. A genome-wide screen identifies genes in rhizosphere-associated *Pseudomonas* required to evade plant defenses. MBio 9:e00433-18.

20. Ueda A, Wood TK. 2010. Tyrosine phosphatase TpbA of Pseudomonas aeruginosa controls extracellular DNA via cyclic diguanylic acid concentrations. Environ Microbiol Rep 2:449–455.

21. Carman GM, Han GS. 2009. Phosphatidic acid phosphatase, a key enzyme in the regulation of lipid synthesis. J Biol Chem 284:2593–2597.

22. Wiesmann CL, Zhang Y, Alford M, Thoms D, Dostert M, Wilson AJ, Pletzer D, Hancock REW, Haney CH. 2021. The *ColR/S* two-component system is a conserved determinant of host association across *Pseudomonas* species. bioRxiv https://doi.org/10.1101/2021.12.14.472530.

23. Miller CD, Kim YC, Anderson AJ. 1997. Cloning and mutational analysis of the gene for the stationary-phase inducible catalase (*catC*) from *Pseudomonas putida*. J Bacteriol 179:5241–5245.
