# Supplementary material for: Commensal Pseudomonas fluorescens Strains Protect Arabidopsis from Closely Related Pseudomonas Pathogens in a Colonization-Dependent Manner
Source: mBio. 2022 Feb 1;13(1):e02892-21. doi: 10.1128/mbio.02892-21 (PMC8805031; doi:10.1128/mbio.02892-21)
Supplement: FIG S6 [file mbio.02892-21-sf006.pdf]

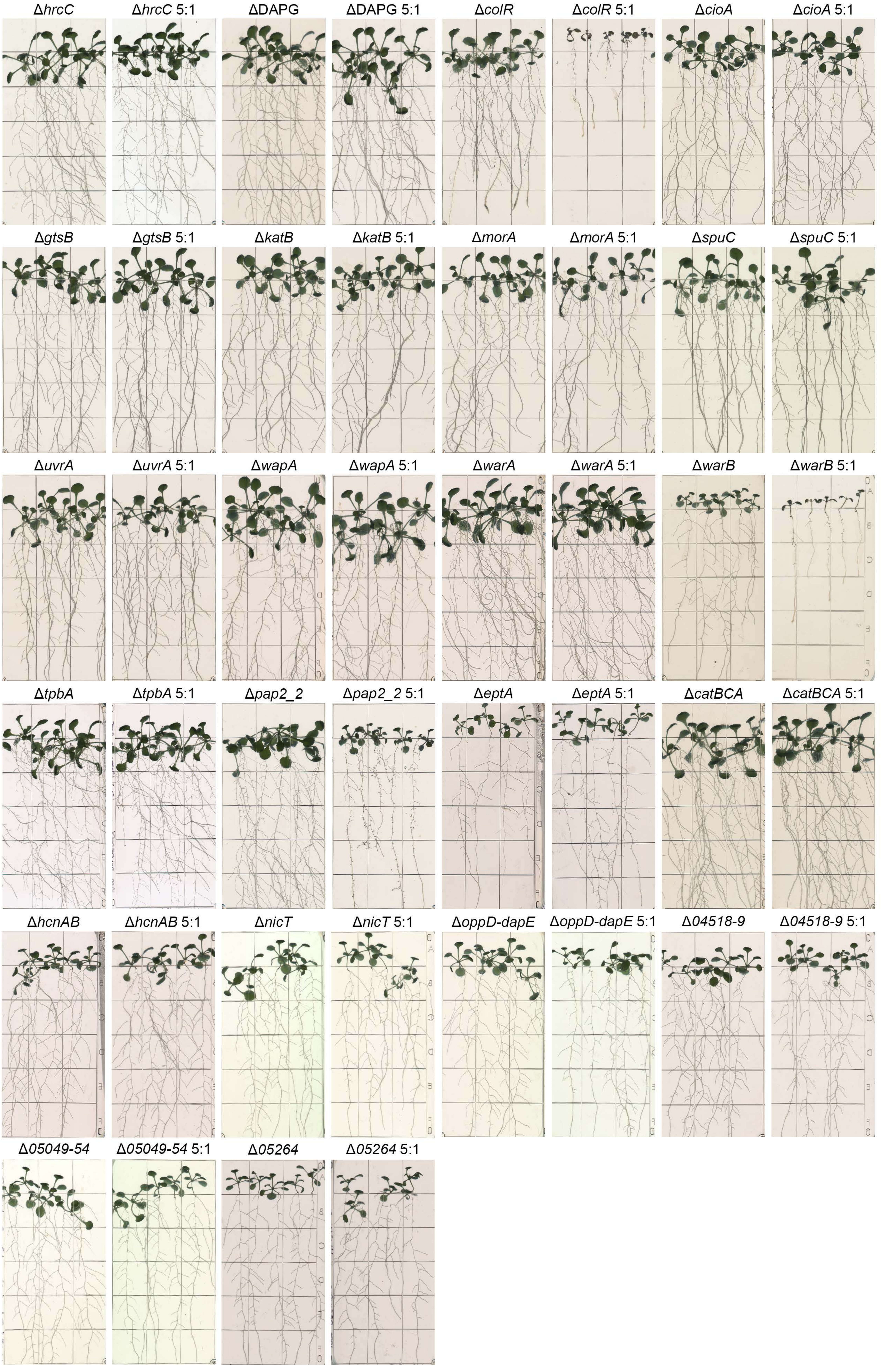
ΔhrcC
ΔhrcC 5:1
ΔDAPG
ΔDAPG 5:1
ΔcolR
ΔcolR 5:1
ΔcioA
ΔcioA 5:1
ΔgtsB
ΔgtsB 5:1
ΔkatB
ΔkatB 5:1
ΔmorA
ΔmorA 5:1
ΔspuC
ΔspuC 5:1
ΔuvrA
ΔuvrA 5:1
ΔwapA
ΔwapA 5:1
ΔwarA
ΔwarA 5:1
ΔwarB
ΔwarB 5:1
ΔtpbA
ΔtpbA 5:1
Δpap2_2
Δpap2_2 5:1
ΔeptA
ΔeptA 5:1
ΔcatBCA
ΔcatBCA 5:1
ΔhcnAB
ΔhcnAB 5:1
ΔnicT
ΔnicT 5:1
ΔoppD-dapE
ΔoppD-dapE 5:1
Δ04518-9
Δ04518-9 5:1
Δ05049-54
Δ05049-54 5:1
Δ05264
Δ05264 5:1
